# Exploring Differences in Pharmacometrics of Rabeprazole between Genders via Population Pharmacokinetic–Pharmacodynamic Modeling

**DOI:** 10.3390/biomedicines11113021

**Published:** 2023-11-10

**Authors:** Seung-Hyun Jeong, Ji-Hun Jang, Yong-Bok Lee

**Affiliations:** 1 College of Pharmacy, Sunchon National University, 255 Jungang-ro, Suncheon-si 57922, Republic of Korea; jeongsh@scnu.ac.kr (S.-H.J.); jangji0121@naver.com (J.-H.J.); 2 College of Pharmacy and Research Institute of Life and Pharmaceutical Sciences, Sunchon National University, Suncheon-si 57922, Republic of Korea; 3 College of Pharmacy, Chonnam National University, 77 Yongbong-ro, Buk-gu, Gwangju 61186, Republic of Korea

**Keywords:** rabeprazole, gender differences, population pharmacokinetic modeling, pharmacodynamics, absorption phase, gastric pH

## Abstract

Rabeprazole is a proton pump inhibitor that inhibits gastric acid production and increases gastric pH; it is widely used clinically as a treatment option for gastritis and gastric ulcers. However, information on the inter-individual variability of rabeprazole pharmacometrics, which is a key element in establishing its scientific clinical use, is still lacking. Particularly, the differences in pharmacokinetics between genders and the degree of variation in pharmacodynamics have not been clearly identified. Thus, the main purpose of this study was to explore any differences in rabeprazole pharmacokinetics between genders and to quantitatively predict and compare the effects of any differences in pharmacokinetics between genders on known pharmacodynamics using population pharmacokinetic–pharmacodynamic modeling. To compare pharmacokinetics and modeling data between genders, bioequivalence results were used simultaneously on healthy Korean men and women using the physiological and biochemical parameters derived from each individual. Pharmacodynamic modeling was performed based on the data of previously reported gastric pH changes in response to rabeprazole plasma concentrations, which was co-linked to the central compartmental bioavailable concentration in the population pharmacokinetic model. There was no significant difference in the level of rabeprazole exposure and elimination of plasma between genders following oral administration of 10 mg enteric-coated rabeprazole tablets; however, there was a clear delay in absorption in women compared to men. Additionally, a comparison of pharmacokinetic parameters normalized to body weight between genders showed that the maximum plasma concentrations were significantly higher in women than in men, again suggesting gender differences in rabeprazole absorption. The population pharmacokinetic profiles for rabeprazole were described using a three-sequential multi-absorption with lag time (T_lag_) two-compartment model, whereas body surface area and gender were explored as effective covariates for absorption rate constant and T_lag_, respectively. The effect of increased gastric pH due to plasma exposure to rabeprazole was explained using the Sigmoid Emax model, with the baseline as a direct response. The significantly longer rabeprazole T_lag_ in females delayed the onset of an effect by an average of 1.58 times (2.02–3.20 h), yet the overall and maximum effects did not cause a significant difference within 15%. In the relative comparison of the overall efficacy of rabeprazole enteric-coated tablet administration between genders, it was predicted based on the model that males would have higher efficacy. This study will be very useful in broadening the perspective of interpreting drug diversity between individuals and narrowing the gap in knowledge related to scientific precision medicine by presenting new information on gender differences in rabeprazole pharmacometrics that had not been previously identified.

## 1. Introduction

Rabeprazole is a proton pump inhibitor (PPI) that inactivates H^+^/K^+^-ATPase in gastric parietal cells, thereby inhibiting the production of gastric acid and increasing gastric pH [1]. Therefore, rabeprazole is frequently used clinically to treat various diseases related to the stomach. Moreover, it is a preferred option for the treatment of gastritis and gastric ulcers, caused by excessive gastric acid production [2], the alleviation of gastroesophageal reflux disease [3], and heartburn symptoms [4], as well as eradicating *Helicobacter pylori* [5]. It has been reported that rabeprazole is extensively metabolized in the body through the cytochrome P450 (CYP) enzyme system in the liver as well as the non-enzymatic system [6]. In the CYP enzyme system, CYP3A4 and CYP2C19 are reported as major sub-types, which convert rabeprazole into sulfone and its demethylated forms, respectively. It is known that thioethers are mainly formed by the non-enzymatic system, which also contributes more significantly to metabolism than the CYP enzyme system [5,6,7]. Therefore, among drugs classified as PPI (omeprazole, esomeprazole, pantoprazole, and lansoprazole), rabeprazole, which is relatively the least affected by CYP polymorphisms, has the advantage of being applicable in clinical practice by minimizing genetic effects between individuals [8]. The in vivo metabolites of rabeprazole are mainly excreted through the kidneys and urine [9]. However, according to a past report [9], the pharmacokinetic profiles of rabeprazole were not significantly altered in patients with renal dysfunction and required maintenance hemodialysis, and no dose modification was required. Rather, changes in the pharmacokinetic profile of rabeprazole were observed in the chronic cirrhosis patient group, although no significant correlation was confirmed with any clinical side effects [9]. Nevertheless, it has been suggested that careful judgment and monitoring are needed for administration in patients with severe liver disease [9]. Some past reports [10,11] have suggested that the pharmacokinetic effects of rabeprazole from food may not be significant. According to a past report [11], no clinically relevant differences were detected in the area under the curve for the plasma concentration–time profiles (AUC), maximum plasma concentration (C_max_), and half-life (T_1/2_) of rabeprazole between fasted and fed conditions. It only caused delayed absorption as time to reach maximum plasma concentration (T_max_) increased with diet. The results suggested that concomitant intake of rabeprazole with a meal may slow down the rate, but not the extend of rabeprazole tablet absorption [10,11]. Additionally, no significant differences were identified in comparisons of the efficacy (measured by changes in 24 h intragastric pH and percentage time at pH > 4) of rabeprazole tablets depending on whether they were taken with food or not [12]. Reports of information on drug interactions with rabeprazole are very limited [13]. According to past reports [13,14], rabeprazole has a lower interaction with CYP than the PPI class omeprazole, suggesting that CYP mediated drug interactions will generally not be significant.

PPIs, including rabeprazole, have a common acid-labile, and many of them are formulated into enteric-coated tablets and applied clinically to prevent decomposition in the stomach, while also increasing its absorption in the small intestine [15,16]. Enteric-coated tablets consist of a drug-loaded core, which is externally coated in polymers (such as polymethacrylates, cellulose acetate phthalate, and hydroxy propyl methyl cellulose phthalate) that have an anti-dissolving function in the acidic environment of the stomach [17]. Therefore, after oral administration, enteric-coated rabeprazole moves to the upper small intestine with minimal drug loss occurring in the stomach, which then disintegrates, dissolves, and is absorbed into the systemic circulation.

The common side effects of rabeprazole include headaches, diarrhea, constipation, nausea and vomiting, feeling dizzy or tired, and sore throat [18]. Additionally, depending on long-term usage of 3–12 months or more, the possibility of hypomagnesemia, bone fractures, gut infections, and vitamin B_12_ deficiency is possible [18]. However, the short- to medium-term use of rabeprazole has a high relative safety margin compared to other high-risk drugs [19,20]. According to one clinical report [21], the daily oral administration of rabeprazole (10 mg) for 104 weeks was judged to be relatively safe, with significant therapeutic effects and no major side effects observed. In terms of drug efficacy, maintenance above the appropriate effective concentration is considered a very important clinical point because drug therapy that extends the duration below the effective concentration significantly increases the time required for a complete cure and is closely related to a high treatment failure rate [22].

Despite frequent clinical applications, information on the quantitative pharmacokinetics and pharmacodynamics of rabeprazole among subjects is still lacking. Simply, pharmacometrics data for establishing a scientific dosage and predicting results were limited to only a few elements (such as CYP2C19), thereby showing a clear knowledge gap in currently integrated pharmacometrics analyses. Past reports [22,23] focused on changes in rabeprazole pharmacokinetics due to CYP2C19 genetic polymorphisms (between extensive and poor metabolizers) and overlooked possible pharmacokinetic differences between genders. Generally, an accepted scenario is that physiological differences will inevitably occur between genders and can represent a major factor that can cause significant differences in pharmacokinetics when exposed to drug formulations of the same content, leading to differences in pharmacodynamics [24,25]. Therefore, previous studies analyzed the differences in pharmacokinetics and pharmacodynamics between genders for drugs actively applied in clinical practice. For example, a significant difference was confirmed between genders in the clearance of zolpidem in vivo, meaning there was a difference in drug efficacy; thus, the application of different doses was suggested between genders [26]. However, to confirm any differences in pharmacokinetics between genders, barriers, such as designing clinical trials, which consider gender differences and the difficulty of interpreting comprehensive results, mean studies on pharmacometrics differences between genders are limited.

The main purpose of this study was to explore gender differences in the pharmacokinetics of rabeprazole that had not been previously identified and to quantitatively interpret the resulting pharmacodynamic differences. In addition, using rabeprazole, which is known to have a higher contribution to the non-enzymatic system than to the CYP enzyme system [5,6,7], we sought to further discover the relationships relating to the physiological and biochemical factors in interpreting the pharmacokinetic variabilities between individuals. Additionally, the resulting efficacy and safety considerations based on the interpretation of the pharmacokinetic diversity of rabeprazole between genders and individuals will be of great clinical interest, and any additional discovery of quantitative predictive factors will correspond to the progressive process of accelerating precision medicine [27,28]. The pharmacokinetic analysis and pharmacometrics modeling results for rabeprazole between genders presented in this study represent particularly useful data for advancing scientific clinical therapy settings that consider the inter-individual variability (IIV) of rabeprazole.

## 2. Materials and Methods

### 2.1. Overview of the Research Approach

This study was conducted in five major steps. First, pharmacokinetic comparisons between genders were performed via non-compartmental analysis (NCA) and graphical profiling. Data for pharmacokinetic comparisons were derived from the bioequivalence results, whereby gender was considered from the clinical design stage. Second, a correlation analysis was performed between the pharmacokinetic parameters and the physiological and biochemical parameters of each individual. This was performed to pre-emptively screen potential covariates applicable in interpreting rabeprazole pharmacokinetic variabilities within the population. Third, population modeling was performed and verified based on the rabeprazole pharmacokinetic data. Here, the basic model structure was established, which included creating the intra- and inter-individual error models, whereas the covariates were reflected to explain the pharmacokinetic variabilities between individuals; gender was considered as a covariate. Fourth, a pharmacodynamic model was established based on the reported results of changes in gastric pH depending on the rabeprazole plasma concentration. The established pharmacodynamic model was co-linked to the pharmacokinetic model so that the degree of drug efficacy could be simulated according to changes in the rabeprazole plasma concentration. Fifth, a quantitative prediction simulation of pharmacokinetics and drug efficacy was performed in the final established rabeprazole pharmacometrics model by reflecting effective covariates, especially gender.

### 2.2. Comparison of Pharmacokinetics between Genders

Pharmacokinetic results obtained from a clinical study using 10 mg rabeprazole enteric-coated tablets (Genuonesciences, Seoul, Republic of Korea; Lot number: 21026) administered to 24 healthy Korean men and 21 women were compared between genders. A total of 45 subjects completed the clinical trial without side effects, and their demographical information (including physiological and biochemical parameters) is presented in Table S1. Analysis of biochemical parameters was performed on all subjects participating in the clinical trial and the methods are presented in Supplementary Information S1. Information relating to the selection and progress of clinical trial participants is presented in Supplementary Information S2, and information on the clinical trial design and sampling is presented in Supplementary Information S3. The clinical trial protocol was thoroughly reviewed and officially approved (approval number: MB22-002; approved on 15 February 2022) by the Ministry of Food and Drug Safety (Cheongju-si, Republic of Korea). NCA was used to calculate the rabeprazole pharmacokinetic parameters for males and females before a comparison of rabeprazole pharmacokinetics was performed between genders to determine any significant rabeprazole pharmacokinetic parameters in males and females. Pharmacokinetic parameters calculated by NCA were estimated based on plasma concentration values over time after orally administering a 10 mg rabeprazole enteric-coated tablet. Information on the analytical method applied to quantify rabeprazole among plasma samples is briefly presented in Supplementary Information S4, whereas the methods for calculating pharmacokinetic parameters using NCA are presented in Supplementary Information S5. An additional NCA analysis was performed based on the normalized rabeprazole plasma concentration values for body weight, which is one of the major physiological differences between genders that has been frequently applied in past pharmacokinetic comparative studies between genders [29,30,31], and the results were categorized and compared by gender. The significant differences between genders were compared using a two-tailed *t* test, with significance determined at a *p* value of 0.05. Graphically, differences in pharmacokinetics between genders were confirmed by dividing the rabeprazole plasma concentration profile comparisons by gender.

### 2.3. Correlation Analysis

Correlation analysis between rabeprazole pharmacokinetic parameters and the individual physiological and biochemical parameters was performed by heatmap generation using Seaborn, one of the visualization libraries in Python (version 3.12.0). Heatmap generation has the advantage of being able to check the relationship and degree of correlation between the target feature and various independent variables intuitively and quickly, alongside simultaneously screening for all possible elements in the system. Since correlation analysis could only be applied to physiological and biochemical parameters and pharmacokinetic parameter values, which have the characteristics of continuous data, gender comparisons were performed separately in categorical terms. The correlation coefficients (*r*) had absolute values that did not exceed 1 and ranged from −1 to +1. Additionally, if they were greater than +0.3 or less than −0.3, a valid positive or negative correlation, respectively, was assumed. The factors for which valid correlations were detected in the heatmap results were additionally subjected to linear regression analysis, and the significance of the *r* values was re-confirmed based on a *p* value of 0.05.

### 2.4. Population Pharmacokinetic Modeling

Construction and analysis of the population pharmacokinetic model for rabeprazole were performed using a non-linear mixed effects model approach using Phoenix NLME (version 8.4, Pharsight, Certara Inc., Princeton, NJ, USA) software. An estimation of population pharmacokinetic parameters for rabeprazole was performed using the first-order conditional estimates method with extended least squares estimation (with *ŋ*–*ε* interaction). The development of the rabeprazole population pharmacokinetic model was largely performed in two steps. First, a basic structural model was established that could explain the plasma concentration of rabeprazole following its oral administration. This included a number of basic compartments, whether to reflect the lag time (T_lag_) associated with rabeprazole oral absorption, the mechanistic structuring involved in the absorption process (including multiple absorption), or the selection of relevant error models to account for residual and IIV. Thus, in establishing the model, the values obtained using the NCA calculations were reflected as the initial parameter values, which accelerated the convergence of the final parameters and enabled its effective modeling. Second, a search was performed for model-applicable effective covariates relevant to the inter-individual rabeprazole pharmacokinetic variation modeling. This was achieved by applying stepwise candidate covariates to the IIVs of the pharmacokinetic parameters reflected in the model. Gender and individual physiological and biochemical factors were considered potential effective covariates and treated as categorical and continuous data, respectively, before application priorities were determined based on the results of the previous correlation screening. Appropriate models for each step were selected and performed using various statistical significance tools derived from Phoenix NLME, including Akaike’s information criterion (AIC), twice the negative log-likelihood (−2LL), and goodness-of-fit (GOF) plots, whereas the significance of the total number of parameters applied to the model (increased or decreased degrees of freedom) was also considered. Significance was determined using a Chi-square distribution *p* value of 0.05 (for forward selection) and 0.01 (for backward elimination) at −2LL and objective function value (OFV). The adequacy of the rabeprazole population pharmacokinetic model was verified using GOF (including distribution of residuals), visual predictive check (VPC), and bootstrapping processes. Approaches to each tool for model verification are presented in Supplementary Information S6.

### 2.5. Extension to Pharmacodynamic Model

The pharmacodynamic model was established based on rabeprazole plasma concentration-measured drug efficacy information (quantified using Web-plot-digitizer (version 4.6)), according to the rabeprazole exposure [32]. The efficacy of rabeprazole was indicated by changes in gastric pH, which was related to plasma rabeprazole selectively inhibiting H^+^/K^+^-ATPase in gastric parietal cells, thereby increasing intragastric pH. Pharmacodynamic predictions for rabeprazole were achieved by structuring the effect compartment related to the rabeprazole pharmacokinetic profile in the central compartment. Simply, the change in the plasma pharmacokinetic profile over time after administering rabeprazole is directly related to the change in drug efficacy. Therefore, it could be expanded and implemented as a pharmacodynamic model, whereby the time–drug effect after exposure to rabeprazole can be quantified.

### 2.6. Model Simulation

A model simulation was performed to compare changes qualitatively and quantitatively in the pharmacokinetic profile according to the covariates explored in this study, especially the gender factor, and to confirm the resulting pharmacodynamic effects. To perform pharmacodynamic simulations based on the rabeprazole population pharmacokinetic model, the rabeprazole population pharmacokinetic model structure developed and validated in this study was fixed. The parameter values of the model were fixed as the mean values of the group, excluding parameters that were considered to reflect the covariates. Parameters for which covariates were reflected were fixed as the average values of the group, according to changes in covariate values by additionally considering covariate–parameter correlation values. Model simulations were performed by predicting and comparing changes in pharmacokinetic parameter values in response to covariates and resulting pharmacokinetic–pharmacodynamic outputs. The simulation and prediction engines of Phoenix NLME were used in the model simulation process.

## 3. Results

### 3.1. Gender Differences in Rabeprazole Pharmacokinetics

**Figure 1:**
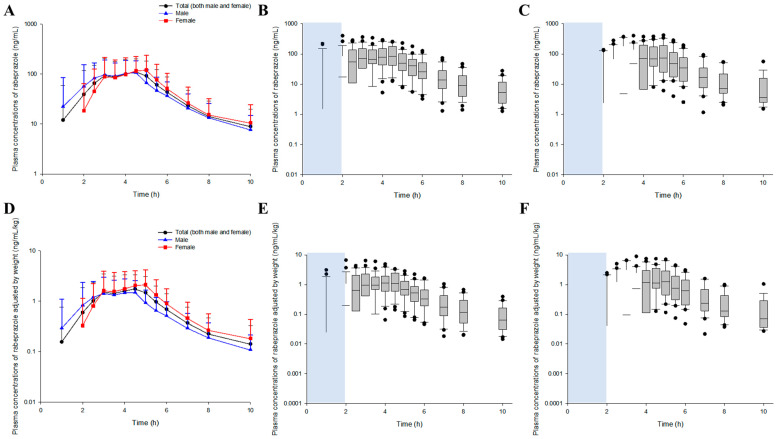
Plasma concentration profiles before (**A**–**C**) and after (**D**–**F**) body weight normalization between genders (**B**,**E**: male; **C**,**F**: female) following oral administration of rabeprazole 10 mg enteric-coated tablets. In graphs (**A**,**D**), observations are presented as mean and standard deviation as dots and upward vertical bars, respectively. (**B**,**C**,**E**,**F**) represent boxplots of plasma concentration values after rabeprazole exposure by time point, and the blue shading in the graph represents the initial absorption phase area from 0 to 2 h after exposure (established to check absorption differences between genders after exposure).

The time–plasma concentration profiles following a single oral administration of a 10 mg rabeprazole enteric-coated tablet are shown in Figure 1. 

Oral absorption of rabeprazole progressed slowly but continuously from 1 h to approximately 3–5 h after administration, potentially due to the characteristics of the enteric-coated tablet formulation, which is designed to not dissolve in the stomach. The rabeprazole plasma concentration profiles showed high IIVs, whereas the relative degree of variation in the initial absorption phase (within 4 h after tablet administration) was the dominant feature across all profile intervals. Comparing pharmacokinetic profiles between genders revealed notable differences in rabeprazole absorption. In males, rabeprazole was detected in plasma 1 h after administration, whereas in females, rabeprazole was not detected in the plasma of any subjects at 1 h post-administration. Unlike the differences in absorption, the profile pattern and rate slope in the elimination phase were similar between genders. 

Table 1 shows the pharmacokinetic parameter values for rabeprazole obtained through NCA. The mean clearance (CL/F) and volume of distribution (V_d_/F) for rabeprazole were high at 25.58 L/h and 53.85 L, respectively, which suggests an extensive elimination and distribution of rabeprazole by the body. Comparatively, significant differences (*p* < 0.05) were confirmed in the pharmacokinetic parameters between genders, with the T_max_ and T_lag_ related to rabeprazole absorption both found to be higher in females. As confirmed in the pharmacokinetic profile comparison (Figure 1), the absorption of rabeprazole enteric-coated tablets was significantly delayed in women compared to men. No significant differences (*p* > 0.05) were found between genders for T_1/2_ and CL/F, thereby suggesting that gender factors are not involved in the elimination of rabeprazole from the body. The fact that there were no significant differences (*p* > 0.05) between genders in the mean residence time (MRT) and V_d_/F implied that gender-related factors were also not significantly involved in the degree of retention and distribution of rabeprazole in the body. Figures S1 and S2 show the boxplot results for both the parameters where significance was not confirmed (*p* > 0.05) and those where it was identified (*p* < 0.05) in the gender-specific comparison of pharmacokinetic parameters obtained using NCA.

Body weight represents a major physiological characteristic that commonly differs between males and females, whereas several past reports [33,34,35] have shown that body weight can affect changes in the pharmacokinetic parameters of a drug, including CL, V_d_, and C_max_. Therefore, to eliminate the inherent influence of body weight factors from the comparison of rabeprazole pharmacokinetics between genders, plasma concentration profiles were normalized to each individual’s body weight, and the pharmacokinetic parameters were estimated. This was because, despite the obvious difference (*p* < 0.05) in body weight between the genders, 10 mg remained of the rabeprazole dose that was administered to all subjects. 

Table 2 shows the pharmacokinetic parameter values calculated using the NCA process based on rabeprazole plasma concentrations following normalization to the individual’s body weight. No significant differences (*p* > 0.05) were identified in the AUC, CL/F (wCL/F), and V_d_/F (wV_d_/F) between genders that were estimated based on the rabeprazole plasma concentrations normalized to body weight. This suggests that even if the weight difference between genders is excluded, the influence of other gender-specific intrinsic factors will not significantly affect the degree of exposure, elimination, and distribution of rabeprazole throughout the body. Alternatively, a significant difference (*p* < 0.05) was confirmed between genders in C_max_ estimations based on the plasma concentration normalized to body weight, where the degree was higher in females, implying that the degree of rabeprazole absorption would be greater in females than in males due to factors other than body weight. Similar to before normalization to body weight (Table 1), significant differences (*p* < 0.05) were confirmed in the T_max_ and T_lag_ values related to rabeprazole absorption, with all values being higher in females. Figure S3 shows the boxplot results for the non-significant factors (*p* > 0.05) in the gender-specific comparison of pharmacokinetic parameters obtained using NCA calculations based on plasma concentrations normalized to body weight. Figure S4 shows a boxplot comparison of C_max_ between genders, which shows significant differences (*p* < 0.05) among the pharmacokinetic parameters calculated based on plasma concentrations before and after normalization to body weight. After the oral administration of a 10 mg rabeprazole enteric-coated tablet, the average C_max_ value was higher in females than in males, although no significant differences were observed between genders due to the inherent body weight factor. This implies that the effect of body weight on changes in C_max_ by rabeprazole could not be ignored.

### 3.2. Population Pharmacokinetic Modeling

The structure of the rabeprazole population pharmacokinetic model can be explained as two compartments, each with three sequential first-order absorption and T_lag_ values. As for the basic compartments, significant model improvements (−2LL reduction of *p* < 0.05 and/or 0.01) were confirmed in two-compartment structures rather than in one, whereas the −2LL increased in three- or more compartment structures alongside the total number of parameters. As a result, the plasma rabeprazole concentration profiles could be explained by its distribution in the central and peripheral compartments with two kinetics in the body. Regarding the delayed absorption pattern of rabeprazole during the absorption phase, several structural absorption compartment models have been attempted, such as the T_lag_ reflection model, the non-sequential two absorption model (having two or more absorption points with consideration of bioavailability), and the sequential absorption model (via the application of two or more absorption rate constant parameters between successive absorption compartments). In addition, mathematical transformation models, such as Weibull absorption, saturation, zero-order, and mean transit time (MTT) have also been attempted. Thus, since T_lag_ showed the largest −2LL reduction, three sequential first-order absorption models that used T_lag_ were selected as the most appropriate absorption model to explain the delayed absorption pattern of rabeprazole. The non-sequential multi-compartments absorption, Weibull, and MTT models also showed a decrease in −2LL compared to the basic model (no T_lag_ with first-order), although the degree was relatively lower than in the sequential first-order absorption model structure with T_lag_. The number of sequential absorption compartments up to 3 was significant (*p* < 0.01) and at the same time significantly improved the GOF plots compared to the basic model; however, from 4 or more, the decrease in −2LL was not significant (*p* > 0.05) compared to the increase in the total number of parameters. The log-additive error model was suitable for use as a residual error model, which when applied, maintained the overall number of parameters and presented a very high degree of reduction in −2LL of 76.93%. Residual error models, such as additive, power, and mixed, significantly increased −2LL rather than the proportional error applied in the basic model. Moreover, even if the −2LL decreased, the magnitude was not significant (*p* > 0.05) or relatively large. The IIVs in the rabeprazole pharmacokinetic parameters were explained by applying an exponential error model. Since step-by-step confirmation illustrated the need to consider IIV in each parameter for model improvement, IIV was considered for central compartment distribution volume (V_c_/F), central compartment clearance (CL_c_/F), first absorption rate constant (K_a1_), second absorption rate constant (K_a2_), third absorption rate constant (K_a3_), and T_lag_. K_a1_, K_a2_, and K_a3_, which were the rate constants between each absorption compartment (dosing depot–depot 1, depot 1–depot 2, depot 2–central compartment) in the multiple sequential absorption of rabeprazole. However, considering IIVs in the peripheral compartment distribution volume (V_p_/F) and peripheral compartment clearance (CL_p_/F) did not significantly improve the model (*p* > 0.05 and/or 0.01 in −2LL reduction) as the number of parameters increased compared to models that did not. An analysis of whether the IIV of the parameters was necessary was conducted by determining the degree of model improvement by sequentially removing the IIV of each parameter, based on the full model in which all the IIVs of the model parameters were considered. 

**Figure 2:**
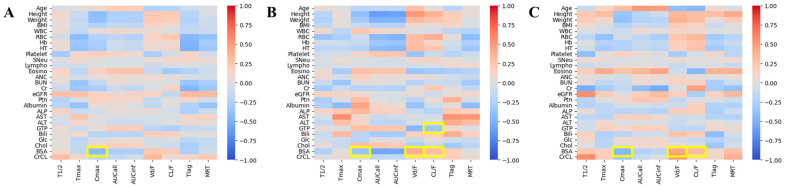
Heatmap results ((**A**) both males and females; (**B**) males; (**C**) females) analyzing the correlation between pharmacokinetic parameter values according to oral administration of 10 mg rabeprazole enteric-coated tablet and biochemical parameters of each individual. Yellow boxes displayed in the heatmap indicate the detection of factors that can reasonably explain the correlation between physiological and biochemical parameters and pharmacokinetic parameters with an absolute correlation coefficient (*r*) of 0.3 or higher. BMI: body mass index; WBC: white blood cell count; RBC: red blood cell count; Hb: hemoglobin; HT: hematocrit; SNeu: Seg-neutrophils; Lympho: lymphocytes; Eosino: eosinophils; ANC: absolute neutrophil count; BUN: blood–urea–nitrogen; Cr: creatinine; eGFR: estimated glomerular filtration rate; Ptn: total protein; ALP: alkaline phosphatase; AST: aspartate transaminase; ALT: alanine transaminase; GTP: gamma-glutamyl transpeptidase; Bili: total bilirubin; Glc: glucose; Chol: total cholesterol; BSA: body surface area; CrCL: creatinine clearance; T_1/2_: half-life; C_max_: maximum plasma concentration; T_max_: time to reach C_max_; AUC_all_: area under the curve from 0 to observed (t) time after administration; AUC_inf_: area under the curve from 0 to infinity time after administration; V_d_: volume of distribution; CL: clearance; F: oral bioavailability; T_lag_: lag time in absorption; MRT: mean residence time.

Table 3 shows a summary of the building procedures used to establish the rabeprazole basic pharmacokinetic structural model. Several physiological and biochemical factors were measured during the clinical trials and gender factors and considered as candidate covariates that could explain the inter-individual pharmacokinetic variabilities of rabeprazole. The prioritized selection of physiological and biochemical factor candidate covariates for model application was performed by NCA and physiological and biochemical factors based on the results of the heatmap continuous variable correlation analysis between pharmacokinetic parameter values. Figure 2 shows the heatmap correlation screening results between the physiological and biochemical factors of each individual and the NCA pharmacokinetic parameter values. 

**Figure 3:**
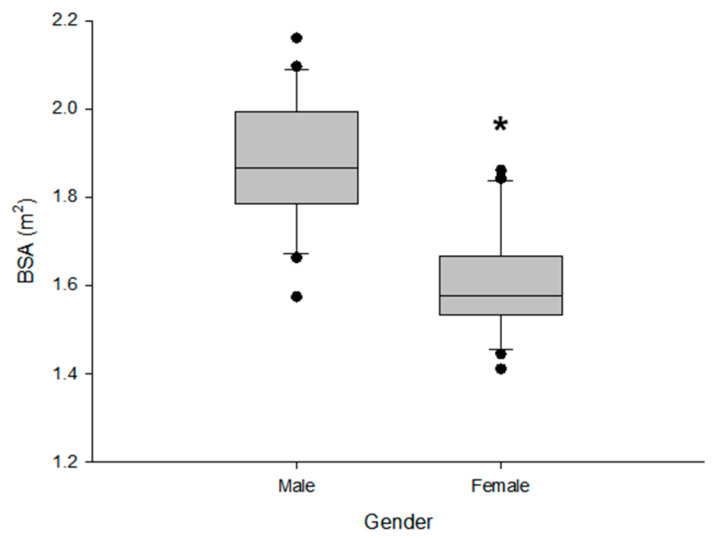
Boxplot results comparing differences in body surface area (BSA) between genders and showing significant correlations with pharmacokinetic parameters identified in the heatmap. * *p* < 0.05 between male parameter values.

For both gender-categorized and non-categorized outcomes, the focus was on factors that could provide a reasonable explanation between the physiological factors and pharmacokinetic parameters and had an absolute *r* of 0.30 or higher. As a result, a common negative correlation was confirmed between body surface area (BSA) and C_max_. Figure 3 shows a comparison of BSA between genders, which showed a common significant correlation (*p* < 0.05) with the pharmacokinetic parameters identified in the heatmap. 

There was a significant difference (*p* < 0.05) in BSA levels between genders, whereby it was lower in females than in males. Nevertheless, when the heatmap was categorized by gender (Figure 2), BSA was commonly significantly correlated with C_max_, suggesting that BSA may affect oral absorption of rabeprazole regardless of gender. Therefore, BSA was selected as a preferential candidate covariate, whereas an attempt was made for it to be reflected in the T_lag_ and rate constants values (K_a1_, K_a2_, and K_a3_) related to rabeprazole absorption. Additionally, attempts were made for other candidate covariates to be reflected in the model through stepwise addition and deletion processes using BSA as a covariate. This process was used to search for a correlation model, in which OFV changes were significant, by sequentially applying or removing candidate covariates in the model parameters for which IIV was considered. Significant correlation was confirmed via forward selection and backward elimination of the covariates in the model parameters, while the *p* values were 0.05 and 0.01, respectively.

Finally, in explaining the inter-individual pharmacokinetic variabilities of rabeprazole, gender, and BSA were considered effective covariates with respect to T_lag_ and K_a3_, respectively. Numerically, significant model improvement (based on the *p* < 0.05 and 0.01 for forward selection and backward elimination) was confirmed by applying gender and BSA as covariates for T_lag_ and K_a3_, respectively, and the GOF plots also showed excellent symmetry for the overall residuals and an appropriate linear correlation between the observed and predicted values. In an attempt to apply BSA as a covariate for V_c_/F and CL_c_/F, the degree of reduction in OFV was more than −3.84, which was suitable for the forward selection criteria (*p* < 0.05) but was not significant in the backward elimination process (*p* > 0.01). Therefore, BSA was not selected as an effective covariate for V_c_/F and CL_c_/F. The gender factor showed a decrease in OFV of −157.97, when reflected as a covariate only in T_lag_ in relation to rabeprazole absorption, whereas there was no significant model improvement in the K_a_ parameters (*p* > 0.05). The covariate reflection of the gender factor in the pharmacokinetic parameters related to rabeprazole body distribution and elimination was not significant in improving the model (*p* > 0.05). 

The steps and results of the covariate reflection for possible factors that could be attempted by prioritizing the established rabeprazole basic population pharmacokinetic model parameters are summarized in Table 4. The structural equations for the final established population pharmacokinetic model on rabeprazole are presented in Supplementary Information S7, and the model parameters and related values are presented in Table 5. 

The coefficient of variation (CV) of typical pharmacokinetic parameter values for K_a1_, K_a2_, K_a3_, V_c_/F, V_p_/F, CL_c_/F, CL_p_/F, and T_lag_ were all within a reasonable agreement of 40% (Table 5). The high estimates of 10.31 and 11.46 L/h for V_c_/F and V_p_/F, respectively, and 25.65 and 5.45 L/h for CL_c_/F and CL_p_/F, respectively, suggested widespread biodistribution and rapid elimination of the exposed rabeprazole in the body. This was consistent with the high mean results of 53.85 L and 25.58 L/h for V_d_/F and CL/F calculated by NCA (Table 1). The positive value for the correlation between T_lag_ and gender implied that the model appropriately explained the pharmacokinetic profiles (Figure 1), which showed a significant absorption delay in females compared to males. The negative correlation value between K_a3_ and BSA meant that K_a3_ increased as BSA decreased. This was interpreted from the faster absorption rate of rabeprazole into plasma that was observed in women than in men via the delayed time point—as shown in the comparison of pharmacokinetic profiles between genders (Figure 1). The reason why plasma concentrations corresponding to raw data, rather than values normalized to body weight, were used in the rabeprazole population pharmacokinetic modeling was to attempt to apply all potential covariates (including body weight and related factors, such as BSA) at the full model level in interpreting rabeprazole pharmacokinetic diversity.

**Figure 4:**
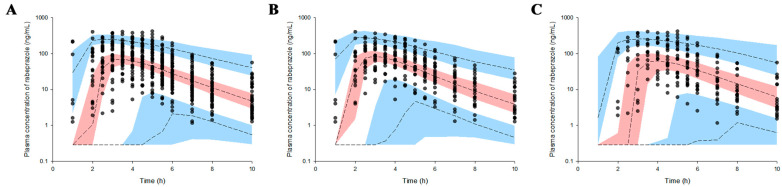
Population pharmacokinetic modeling visual predictive check (VPC) results of observed plasma concentrations following oral administration of 10 mg rabeprazole enteric-coated tablets. Model VPC results are presented separately for the total (**A**), without stratification, and for males (**B**) and females (**C**), separately and stratified by gender. Observed concentrations are depicted by the dots. The 95th, 50th, and 5th percentiles of the predicted concentrations are represented by black dashed lines. The 95% confidence intervals (CIs) for the predicted 5th and 95th percentiles are represented by the blue-shaded regions. The 95% CIs for the predicted 50th percentiles are represented by the red-shaded regions.

The GOF plot results for the rabeprazole population pharmacokinetic model established in this study are presented in Figure S5. Indeed, relatively good agreement was observed between the rabeprazole concentration values in the population or individuals predicted by the population pharmacokinetic model and the experimentally obtained observations. The conditional weighted residuals (CWRES) were well distributed symmetrically with respect to zero. That is, CWRES were well distributed at random without any remarkably specific bias. Further, the CWRES values did not deviate from ±4 at any point in the predicted concentrations or time in the population. the quantile–quantile (QQ) plots of the CWRES components were close to a straight line, meaning the X- and Y-axes were symmetrical (within ±6 ranges). Consequently, the GOF plot results (Figure S5) suggested that the final established population pharmacokinetic model for rabeprazole had no graphically significant problems. Bootstrapping results for the established rabeprazole population pharmacokinetic model are presented in Table 5. All the parameter values estimated in the final model for rabeprazole were within the 95% confidence interval (CI) of the bootstrap analysis results (1000 replicates). Additionally, the model parameter estimates were close to the median estimated by the bootstrap analysis, with the differences within 30%. Therefore, bootstrapping analysis confirmed the robustness and reproducibility of the final established population pharmacokinetic model for rabeprazole. The VPC result of the rabeprazole population pharmacokinetic model is presented in Figure 4. 

Most of the observation values (>90% of all data) associated with the rabeprazole pharmacokinetics were well distributed within the 95% CIs of the predicted values. The VPC results suggested that the rabeprazole population pharmacokinetic model described the overall experimental data relatively well. As a result, the final established population pharmacokinetic model for rabeprazole was at an acceptable level in the overall evaluation results, meaning there were no major problems.

### 3.3. Expansion to the Pharmacodynamic Model

**Figure 5:**
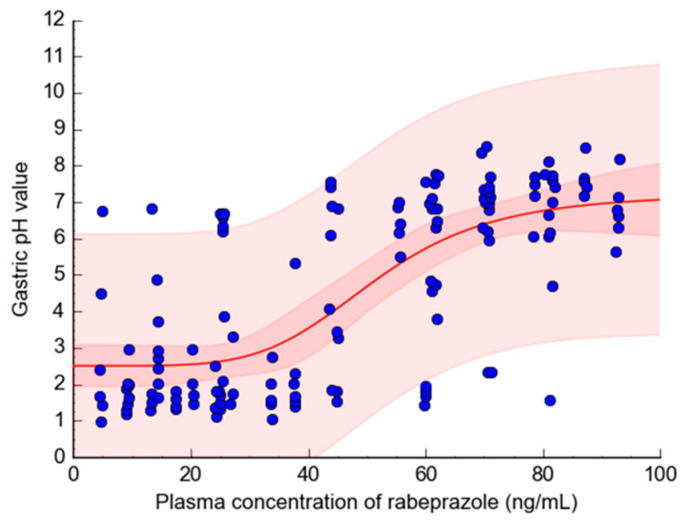
Model fitting result (by applying sigmoid E_max_ model with baseline) of gastric pH values according to rabeprazole plasma concentration. Observed values digitized from the report by Chen et al. (2006) [32] are depicted by the dots. The red line represents the mean values predicted by the model. The dark and light red bands represent the 95% confidence interval and the prediction interval, respectively.

The established and validated population pharmacokinetic model structure and parameter values associated with rabeprazole were fixed as representative values of the population before being expanded into a model to predict the pharmacodynamics of rabeprazole. This was conducted to explore the effect of any differences in the absorption rate of rabeprazole between genders. Pharmacodynamic modeling was performed using the previously reported gastric pH change data [32], according to plasma rabeprazole concentrations after rabeprazole administration, meaning the pharmacodynamic data could be finally explained using the sigmoid E_max_ model with baseline values. Figure 5 shows the graphical results from fitting the sigmoid E_max_ model, with a baseline, to the pharmacodynamic data. 

The observations overlapped well with the overall model-predicted mean values, with more than 90% of all observations included within the 95% CI. In selecting the pharmacodynamic model, several direct and indirect response models were applied sequentially, with the criteria exhibiting the best fit to the observations and reasonable interpretation. During model fitting, the quantitative indices of AIC and −2LL served as the basis for judging objective model suitability. The sigmoid E_max_ model with a baseline showed a high *r* of 0.71 when using the lowest AIC and −2LL values in the models. 

Table 6 shows the formula and configuration parameter values of the rabeprazole pharmacodynamic model established in this study. The relative standard errors (RSEs) of the pharmacodynamic model parameters E_0_, E_max_, and EC_50_ were reasonable values within 20%. Conversely, the high γ RSE of 42.46% was interpreted as being related to the significant inter-individual differences in the degree of drug efficacy in raising gastric pH as the plasma concentration of rabeprazole increased. The E_0_, E_max_, EC_50_, and γ parameters exhibited in the sigmoid E_max_ model with a baseline depict the basal pH effect in the stomach that occurs without rabeprazole, the maximal effect of increasing gastric pH by rabeprazole in plasma, the concentration of rabeprazole in plasma required to achieve half of the E_max_, and the sigmoidicity factor related to the steepness of the profile, respectively.

### 3.4. Exploring Gender Differences in Pharmacometrics Based on Model Simulations

**Figure 6:**
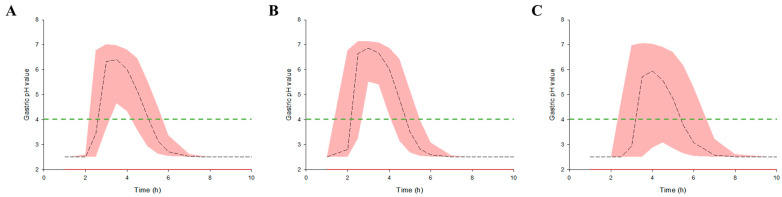
Model prediction results of the gastric pH profiles over time after a single oral exposure to a 10 mg rabeprazole enteric-coated tablet. Prediction results were presented separately for total (**A**) without stratification and for males (**B**) and females (**C**), separately and stratified by gender. The 50th percentiles of the predicted pH values are represented by black dashed lines and the 95% confidence intervals for the predicted 50th percentiles are represented by the red-shaded regions. The green dotted lines in the graph represent the reference value for 4, the pH rise point established as the effective treatment effect of rabeprazole.

Model simulations were performed using numerical changes and reflections of effective covariates in the final established rabeprazole population pharmacokinetic–pharmacodynamic co-linked model. The effective covariates explored during the rabeprazole population pharmacokinetic modeling process were gender and BSA, with gender being categorical and BSA being continuous (reflected as median values for males and females). The observed median BSA values for males and females in the population were 1.87 and 1.58 m^2^, respectively. Figure 6 shows the pharmacodynamic model simulation results according to gender and the BSA reflection in that gender after oral administration of a 10 mg enteric-coated rabeprazole tablet. 

Consistent with the pharmacokinetic prediction based on the population pharmacokinetic model (Figure 4), the increase in gastric pH was delayed in females compared to males. The expected delay in the increased gastric pH effect following oral exposure to rabeprazole in females may be related to the significant T_lag_ identified between genders in the pharmacokinetic model. 

Table 7 shows a quantitative comparison of the gastric pH increase according to gender after oral administration of a 10 mg enteric-coated rabeprazole tablet. The area under the effect curve in the graph relating to the pH change according to rabeprazole exposure time (AUEC) and max effect above pH 4 between genders according to rabeprazole oral exposure did not show much difference at around 15%, although the onset time of the effect was delayed by an average of 1.58 times (2.02–3.20 h) in females. Alternatively, males had an effect duration time that was 1.33 times longer (2.09–2.79 h), on average. As a result, it was suggested that the oral administration of enteric-coated rabeprazole tablets by males may result in a relatively faster increase in gastric pH than in females and that the effect may last for a longer period of time.

## 4. Discussion

The significantly higher absorption delay observed in females could be explained by gender differences in gastric emptying time. Past reports have identified gender as a factor influencing gastric emptying, with slower emptying of both solids and liquids noted in the stomachs of women compared to healthy men [36,37]. In particular, the gastric emptying rate of solid materials was significantly (*p* < 0.05) slower in females than in males [36]. Therefore, it took longer in women for the orally administered rabeprazole enteric-coated tablet to completely pass through the stomach and reach the upper small intestine, where it can be absorbed into the plasma, meaning that the absorption rate-related parameters, T_lag_ and T_max_, were significantly increased. When combining the NCA and population pharmacokinetic modeling results based on pharmacokinetic profiles, significant differences were mainly observed in the pharmacokinetics of rabeprazole between genders and/or individuals in the absorption phase. That is, the onset of rabeprazole absorption into the body was significantly slower in females than in males, and the rate of absorption into plasma at the delayed time point became slower as the BSA increased. The association between BSA and the absorption rate into plasma at delayed time points can be interpreted in relation to the significant differences in BSA levels (*p* < 0.05) between genders. BSA showed a significantly lower value in females than in males, which may have resulted in faster rabeprazole absorption after the delayed time point in females than in males. The correlations between the absorption rate into the plasma at delayed time points and BSA were consistent in the pharmacokinetic profile (Figure 1) and VPC model results (Figure 4), suggesting that the model-based parameter interpretations in this study were performed appropriately. The reason why some points in the GOF plot showed high and low DV (observed plasma concentration, natural log scale, ng/mL) values around the PRED (population-predicted concentration, natural log scale, ng/mL) values of −1 (related to the initial absorption phase in males) and 4–6 (related to the initial absorption phase in females), respectively, may be related to the very large IIV in the oral absorption of rabeprazole enteric-coated tablets. In other words, since significant differences were confirmed in T_lag_ and T_max_ in the intestinal absorption of rabeprazole in both males and females (Table 1), it was interpreted that the difference in absorption between PRED and DV occurred in the gender-integrated model GOF plot. This again suggests that gender provided a suitable explanation for the significant differences observed in this study relating to the absorption of rabeprazole enteric-coated tablets.

This study strongly suggests that there may be notable differences in the variability between genders in gastric emptying times. The fact that plasma concentrations of rabeprazole did not appear in all women within 2 h (Figure 1) clearly demonstrates that the absorption window following enteric-coated tablet administration exists in the small intestine, and implies that the gastric emptying time shows less inter-individual differences. On the other hand, in men, the plasma concentrations of rabeprazole appear 1 h after oral administration and the difference between individuals is large, suggesting that gastric emptying time is relatively less constant and fluctuates more than in women.

The fact that the standard deviations in the pharmacokinetic parameter values presented in Table 1 and Table 2 were not relatively small was interpreted to be due to the large IIV in rabeprazole pharmacokinetics. This is also implied by the wide distribution of plasma concentration values at each sampling point in the pharmacokinetic profiles shown in Figure 1. Therefore, considering the significant pharmacokinetic variability of rabeprazole enteric-coated tablets confirmed through clinical trials, analysis and interpretation of the pharmacokinetic diversity between individuals within the population, as in this study, was very urgent and important.

Table S2 shows the demographic information of healthy Korean subjects who received a single oral dose of 10 mg rabeprazole enteric-coated tablet and the comparisons between genders. As a result of comparing demographic information between genders, significant differences (*p* < 0.05) were confirmed in height, body weight, body mass index (BMI), BSA, red blood cell count, hemoglobin, hematocrit, platelet count, eosinophils, blood–urea–nitrogen (BUN), creatinine, estimated glomerular filtration rate (eGFR), albumin, alkaline phosphatase (ALP), alanine transaminase (ALT), and gamma-glutamyl transpeptidase (GTP). As significant differences between genders have already been noted in height and weight, the significance between genders was also confirmed in BMI and BSA, which are factors calculated based on height and weight. Although the significance between genders was confirmed in some hematological indicators (red blood cell count, hemoglobin, hematocrit, platelet count, and eosinophils), the interpretation of any correlation with pharmacokinetic parameters is limited. In addition, although significance between genders was confirmed in biochemical indicators (BUN, creatinine, eGFR, albumin, ALP, ALT, and GTP), they were not effective in interpreting differences between genders in the final rabeprazole pharmacokinetics. That is, renal and liver function indicators were not reflected as effective covariates in the interpretation of the inter-individual pharmacokinetic variabilities relating to rabeprazole. This suggested that significant differences (*p* < 0.05) in biochemical factors between genders would have an almost negligible effect on rabeprazole pharmacokinetic variability (especially distribution and elimination). Among the various factors identified between genders, body weight normalization pharmacokinetic interpretation, which is implied to be closely related to changes in pharmacokinetic parameters (especially AUC, CL/F, V_d_/F, and C_max_) and has been frequently applied in past pharmacokinetic comparisons between genders [29,30,31], was additionally applied in this study. The reason why C_max_, excluding body weight factors, was significantly higher in women than in men could not be explained because a specific transporter was involved in the absorption of rabeprazole in the intestinal tract, and there were gender differences in the level of expression of that transporter. Therefore, future research to explore the intestinal absorption mechanism and substrate-specific transporter of rabeprazole, which is a knowledge gap at this stage, will be able to clarify these differences in rabeprazole absorption between genders. In addition, significant differences between genders occurred not only in gastric emptying but also in intestinal emptying, so the possibility that this may have affected the absorption of rabeprazole in the intestinal tract cannot be ruled out. In other words, similar to the stomach, emptying of the intestinal tract was slower in females than in males, and as a result, intestinal absorption of enteric-coated tablets progressed more effectively (with long retention in the upper small intestine), which may lead to a relative increase in C_max_. Nevertheless, the fact that no significant difference between genders was identified in AUC (Table 1 and Table 2) could be interpreted as a possibility that it is related to the wide and extensive absorption of rabeprazole in the intestine (considering the absorption phase that lasts for approximately 4–5 h after oral administration as shown in Figure 1).

The reason why sequential multiple absorption was suitable for the absorption phase structure of the rabeprazole population pharmacokinetic model established in this study was probably related to the high variability between individuals in rabeprazole absorption in the intestinal tract (including gastric emptying time). This is because reflecting the rate constants between each compartment along with multiple absorption compartments would have been effective in modeling and explaining the IIV (comprehensive variations in absorption delay and rate) in rabeprazole oral absorption. In addition, the model structure that considers IIV for each rate constant between multiple absorption compartments may have served as a suitable factor for explaining the multivariable absorption phase associated with rabeprazole enteric-coated tablets. The sequential multiple absorption model structure has relatively higher flexibility than general one-compartmental or parameterizing absorption models, thereby allowing IIV to be applied consistently to pharmacokinetic parameters related to the absorption phase.

The pharmacodynamic rabeprazole model established in this study relies on the pharmacokinetic rabeprazole profile in plasma and is limited since it does not reflect the separate covariates in the drug efficacy variability between individuals. As shown in the established fitting results of the rabeprazole pharmacodynamic model (Figure 5) based on a previous report [32], it is assumed that the degree of change in the gastric pH varies greatly between individuals depending on the concentration of rabeprazole in the plasma. Therefore, further exploration of effective covariates that can explain the IIV related to the rabeprazole drug response will be necessary in the future. For example, even if the plasma concentration of rabeprazole remains the same, there may be differences in the response sensitivity between individuals due to genetic or physiological factors. Nevertheless, the significance of this study is that it is the first to explore differences in pharmacokinetics and pharmacodynamics for rabeprazole between genders using a pharmacometrics model, which has not been previously reported. The pharmacometrics modeling approaches applied in this study will be very useful in improving and developing the model in future studies (such as additional discovery of covariates and large-scale clinical trials) and will enable successful research results. The reason why the direct response model of the sigmoid E_max_ with baseline was suitable as the pharmacodynamic model for rabeprazole in this study may be related to the faster gastric acid secretion inhibition effect by rabeprazole compared to other PPI drugs [38]. Additionally, the rapid and widespread distribution (particularly high uptake in the stomach) characteristics of rabeprazole in vivo [39] may also be relevant. In other words, a direct response between pharmacokinetics and pharmacodynamics could have been reasonably established by the mechanism through which plasma rabeprazole reaches the gastric parietal cells almost simultaneously and rapidly increases gastric pH by inhibiting H^+^/K^+^-ATPase.

Among PPIs, the rabeprazole pharmacokinetics are less affected by CYP polymorphisms, while rabeprazole is a drug that mostly functions through the non-enzymatic system, meaning that the influence of the CYP2C19 genotype on its pharmacodynamics is reported to be independent [38]. Nevertheless, some past reports [22,23] have shown that the in vivo clearance of rabeprazole was affected by CYP2C19 genetic polymorphisms, and the level of plasma exposure tended to be lower in extensive metabolizers (carriers of *1 and/or *17 alleles) compared to poor metabolizers (carriers of *2 and/or *3 alleles). Therefore, it will be necessary to conduct clinical pharmacometrics research in the future that comprehensively considers the clearance impact due to the inter-individual CYP2C19 polymorphic factor in addition to the absorption influence by the gender factor explored in this study. Through this, more information on the pharmacometrics associated with rabeprazole will be accumulated, which can maximize the clinical predictive power.

A past report suggested changes in rabeprazole pharmacokinetics and the need for subsequent monitoring in patients with severe liver disease [9]. In this study, it was not possible to explore significant correlations or reflect covariates between clinical biochemical indicators and pharmacokinetic parameter values related to liver function. This suggested that within the relatively normal liver function parameters in a healthy adult population, the effect on changes in the pharmacokinetics of rabeprazole would be almost negligible. In other words, this study strongly suggests that the administration of rabeprazole in general patient groups (complaining only of symptoms caused by hypersecretion of gastric acid) without major liver function problems can be performed without considering differences in liver function between individuals. If additional model analysis is conducted in the future, including specific patient groups such as cirrhosis with significantly reduced liver function, the possibility that the measured liver function indicators will be explored as effective covariates of pharmacokinetic model parameters cannot be ruled out. In this study, the failure to explore significant correlations or reflect covariates between clinical biochemical indicators and pharmacokinetic parameter values related to renal function was consistent with the fact that there were no significant changes in rabeprazole pharmacokinetics, even in the group of patients with severe renal failure [9]. Therefore, it was implied that considering gender or inter-individual differences in renal function would not be important in the clinical application of rabeprazole.

## 5. Conclusions

To explore the effect of pharmacokinetic differences in rabeprazole absorption rate between genders on drug efficacy, an attempt was made to expand the co-linkage of the population pharmacokinetic model into a pharmacodynamic model. Thus, this study performed the following integrated pharmacokinetic–pharmacodynamic analysis: After oral administration of rabeprazole enteric-coated tablets in males with a relatively fast gastric emptying time, the tablets rapidly migrated to the upper small intestine, and absorption of rabeprazole into plasma could have occurred within the intestinal tract for a longer period of time. This may have led to a rapid onset of drug efficacy by increasing gastric pH in males and maintaining the drug effect for a longer period of time. This study strongly suggests that clear differences exist in the absorption of rabeprazole pharmacokinetics between genders, which may, consequently, affect the drug onset time. This study presents a very useful perspective on scientific individualized drug therapy and precision medicine for rabeprazole using a quantitative pharmacometrics approach.

## Figures and Tables

**Table 1:** Pharmacokinetic parameter values for males and females following oral administration of 10 mg rabeprazole enteric-coated tablets.

Parameters	Total (*n* = 45)	Male (*n* = 24)	Female (*n* = 21)
AUC_all_ (ng·h/mL)	432.32 ± 166.23	439.23 ± 161.16	424.42 ± 175.50
AUC_inf_ (ng·h/mL)	456.11 ± 174.04	458.78 ± 171.73	453.05 ± 180.84
AUC_extrap_ (%)	5.07 ± 9.42	3.84 ± 4.01	4.38 ± 7.82
CL/F (L/h)	25.58 ± 11.33	25.40 ± 12.23	25.78 ± 10.51
C_max_ (ng/mL)	236.57 ± 97.86	216.19 ± 79.61	259.86 ± 112.74
T_1/2_ (h)	1.55 ± 0.54	1.60 ± 0.44	1.48 ± 0.64
MRT (h)	4.79 ± 1.70	4.40 ± 1.36	5.25 ± 1.97
T_max_ (h)	3.71 ± 1.22	3.38 ± 1.21	4.10 ± 1.15 *
T_lag_ (h)	1.84 ± 1.33	1.25 ± 1.13	2.52 ± 1.23 *
V_d_/F (L)	53.85 ± 27.34	53.46 ± 12.83	54.29 ± 38.14

AUC_all_: area under the curve from 0 to observed (t) time after administration; AUC_inf_: area under the curve from 0 to infinity time after administration; AUC_extrap_: fraction of AUC_inf_ and AUC_all_; CL: clearance; C_max_: maximum plasma concentration; T_1/2_: half-life; MRT: mean residence time; T_max_: time to reach C_max_; T_lag_: lag time in absorption; V_d_: volume of distribution; F: oral bioavailability. * *p* < 0.05 between male pharmacokinetic parameters. Parameter values are presented as mean ± standard deviation.

**Table 2:** Calculated pharmacokinetic parameter values in males and females following oral administration of 10 mg rabeprazole enteric-coated tablet based on plasma concentrations normalized by body weight.

Parameters	Total (*n* = 45)	Male (*n* = 24)	Female (*n* = 21)
AUC_all_ (ng·h/mL/kg)	6.80 ± 3.19	6.19 ± 3.01	7.48 ± 3.32
AUC_inf_ (ng·h/mL/kg)	7.17 ± 3.34	6.47 ± 3.18	7.98 ± 3.42
wCL/F (L/h)	1730.05 ± 881.19	1909.93 ± 965.32	1524.47 ± 743.95
C_max_ (ng/mL/kg)	3.77 ± 2.01	3.02 ± 1.40	4.63 ± 2.28 *
wV_d_/F (L)	3659.71 ± 2075.45	3998.25 ± 1171.40	3272.81 ± 2757.49

AUC_all_: area under the curve from 0 to observed (t) time after administration; AUC_inf_: area under the curve from 0 to infinity time after administration; CL: clearance; C_max_: maximum plasma concentration; V_d_: volume of distribution; F: oral bioavailability. * *p* < 0.05 between male pharmacokinetic parameters. Parameter values are presented as mean ± standard deviation. wCL/F and wV_d_/F refer to the estimated clearance and volume distribution based on plasma rabeprazole concentrations normalized to body weight, respectively.

**Table 3:** Summary of building step procedure results for establishing a basic pharmacokinetic structural model for rabeprazole.

Model	Description	nParameter ^a^	Twice the Negative Log-Likelihood (−2LL)	Akaike’s Information Criterion (AIC)	Δ-2LL ^b^	ΔAIC ^c^	Compared Model
Compartment disposition model							
01	1-compartment	7	6632.06	6646.06	- ^d^	- ^d^	- ^d^
02 *	2-compartment	11	6532.11	6554.11	−99.95	−91.95	01
03	3-compartment	15	6632.11	6662.11	100.00	108.00	02
Absorption model							
02	No lag time (T_lag_) with first order	11	6532.11	6554.11	- ^d^	- ^d^	- ^d^
02-01	Add T_lag_ with first order	13	5305.25	5331.25	−1226.86	−1222.86	02
02-02	Add T_lag_ with sequential two absorption compartment **	15	5124.48	5154.48	−180.77	−176.77	02-01
02-03 *	Add T_lag_ with sequential three absorption compartment **	17	4895.32	4929.32	−409.93	−401.93	02-01
02-04	Add T_lag_ with sequential four absorption compartment **	19	4894.20	4932.20	−1.12	2.88	02-03
02-05	Add T_lag_ with sequential five absorption compartment **	21	4924.15	4966.15	28.83	36.83	02-03
02-06	Add T_lag_ with non-sequential two absorption compartment **	17	5274.37	5308.37	−30.88	−22.88	02-01
02-07	Add T_lag_ with non-sequential three absorption compartment **	19	6439.96	6477.96	1134.71	1146.71	02-01
02-08	Weibull	13	5811.24	5837.24	−720.87	−716.87	02
02-09	Two-function Weibull	19	5800.40	5838.40	−731.71	−715.71	02
02-10	Saturation (Michaelis–Menten kinetic type)	13	6605.69	6631.69	73.58	77.58	02
02-11	Zero-order	11	6612.52	6634.52	80.41	80.41	02
02-12	Mean transit time (MTT) **	15	5401.43	5431.43	−1130.68	−1122.68	02
02-13	Add T_lag_ with MTT **	17	4920.56	4954.56	−384.69	−376.69	02-01
Residual error model							
02-03	Proportional	17	4895.32	4929.32	- ^d^	- ^d^	- ^d^
02-03-01	Additive	17	5124.64	5158.64	229.32	229.32	02-03
02-03-02 *	Log additive	17	1129.25	1163.25	−3766.07	−3766.07	02-03
02-03-03	Mixed	18	4892.35	4928.35	−2.97	−0.97	02-03
02-03-04	Power	17	4884.53	4918.53	−10.79	−10.79	02-03
Inter-individual variability (IIV) model							
02-03-02-01	Remove IIV of central compartment distribution volume (V_c_/F)	16	1424.10	1456.10	294.85	292.85	02-03-02
02-03-02-02	Remove IIV of central compartment clearance (CL_c_/F)	16	1422.58	1454.58	293.33	291.33	02-03-02
02-03-02-03	Remove IIV of peripheral compartment distribution volume (V_p_/F)	16	1115.52	1147.52	−13.73	−15.73	02-03-02
02-03-02-04	Remove IIV of peripheral compartment clearance (CL_p_/F)	16	1114.86	1146.86	−14.39	−16.39	02-03-02
02-03-02-05	Remove IIV of first absorption rate constant (K_a1_) ***	16	1153.43	1185.43	24.18	22.18	02-03-02
02-03-02-06	Remove IIV of second absorption rate constant (K_a2_) ***	16	1154.22	1186.22	24.97	22.97	02-03-02
02-03-02-07	Remove IIV of third absorption rate constant (K_a3_) ***	16	1405.59	1437.59	276.34	274.34	02-03-02
02-03-02-08	Remove IIV of T_lag_	16	1467.88	1499.88	338.63	336.63	02-03-02
02-03-02-09 *	Remove IIV of V_p_/F and CL_p_/F	15	1112.35	1142.35	−2.51	−4.51	02-03-02-04

^a^ nParameter means the total number of parameters applied to the model. ^b^ means the change value of twice the negative log-likelihood according to the comparison between models. ^c^ means the change value of Akaike’s information criterion according to the comparison between models. ^d^ not applicable. * means the model selected in each step; ** means a kind of multiple absorption compartment model; *** K_a1_, K_a2_, and K_a3_ refer to the rate constants between each absorption compartment (K_a1_: dosing depot–depot 1, K_a2_: depot 1–depot 2, K_a3_: depot 2–central compartment) in the multiple sequential absorption of rabeprazole.

**Table 4:** Summary of stepwise selection results for potential covariates (considered as preferential reflection) in the population pharmacokinetic model of rabeprazole.

Model	Description	Objective Function Value (OFV)	△OFV ^a^	Compared Model	nParameter ^b^
1	Base model ^c^	1112.35	- ^d^	- ^d^	15
2	Gender on first absorption rate constant (K_a1_) ***	1113.05	0.70	Base model	16
3	Gender on second absorption rate constant (K_a2_) ***	1112.76	0.41	Base model	16
4	Gender on third absorption rate constant (K_a3_) ***	1110.59	−1.76	Base model	16
5	Gender on lag time (T_lag_)	954.38	−157.97	Base model	16
6	Gender on central compartment distribution volume (V_c_/F)	1110.60	−1.75	Base model	16
7	Gender on central compartment clearance (CL_c_/F)	1110.48	−1.87	Base model	16
8	Body surface area (BSA) on K_a1_ ***	1113.22	0.87	Base model	16
9	BSA on K_a2_ ***	1113.14	0.79	Base model	16
10	BSA on K_a3_ ***	964.35	−148.00	Base model	16
11	BSA on T_lag_	1111.95	−0.40	Base model	16
12	BSA on V_c_/F **	1108.40	−3.95	Base model	16
13	BSA on CL_c_/F **	1108.37	−3.98	Base model	16
14 *	Gender on T_lag_ and BSA on K_a3_ ***	921.86	−32.52	Model 5	17
15	Gamma-glutamyl transpeptidase (GTP) on V_c_/F	1113.09	0.74	Base model	16
16	GTP on CL_c_/F	1112.17	−0.18	Base model	16
17	Body mass index (BMI) on V_c_/F	1109.24	−3.11	Base model	16
18	BMI on CL_c_/F	1109.27	−3.08	Base model	16
19	Creatinine clearance (CrCL) on K_a1_ ***	1114.62	2.27	Base model	16
20	CrCL on K_a2_ ***	1113.81	1.46	Base model	16
21	CrCL on K_a3_ ***	1111.84	−0.51	Base model	16

^a^ means the change in objective function value according to the comparison between models. ^b^ nParameter means the total number of parameters applied to the model. ^c^ base model: 2-compartment disposition, first-order sequential three absorption compartments with T_lag_, log-additive residual error, no consideration of inter-individual variability in peripheral compartment distribution volume (V_p_/F) and clearance (CL_p_/F). ^d^ not applicable. * means the final selected model (via the forward selection of 0.05 *p* value and backward elimination of 0.01 *p* value); ** means that it was suitable for the forward selection criteria but not for the backward elimination process; *** K_a1_, K_a2_, and K_a3_ refer to the rate constants between each absorption compartment (K_a1_: dosing depot–depot 1, K_a2_: depot 1–depot 2, K_a3_: depot 2–central compartment) in the multiple sequential absorption of rabeprazole.

**Table 5:** Parameter values and bootstrap results for the final established population pharmacokinetic model for rabeprazole.

Parameter	Final Model			Bootstrap (*n* = 1000)	
	Estimate	Standard Error	Coefficient of Variation (%)	Median	95% Confidence Interval
tvV_c_/F (L) ^a^	10.31	1.85	17.90	10.06	6.83–14.37
tvCL_c_/F (L/h) ^a^	25.65	1.36	5.30	25.46	23.26–28.03
tvV_p_/F (L) ^a^	11.46	1.20	10.49	11.30	9.43–13.94
tvCL_p_/F (L/h) ^a^	5.45	0.68	12.54	5.38	4.02–6.88
tvK_a1_ (1/h) ^a^	1.91	0.40	21.17	1.89	1.37–3.11
tvK_a2_ (1/h) ^a^	2.43	0.93	38.40	2.03	1.66–4.59
tvK_a3_ (1/h) ^a^	3.34	0.82	24.44	3.26	1.97–5.16
tvT_lag_ (h) ^a^	1.56	0.21	13.37	1.55	1.18–1.98
dT_lag_dGender ^b^	0.73	0.25	34.57	0.67	0.32–1.24
dK_a3_dBSA ^c^	−1.11	0.48	43.24	−0.95	−1.89–−0.01
ε	0.37	0.10	26.38	0.35	0.24–0.58
ω^2^_Vc/F_	2.04	0.61	30.01	1.55	0.35–2.75
ω^2^_CLc/F_	0.14	0.04	25.16	0.14	0.07–0.20
ω^2^_Ka1_	0.00	0.00	28.48	0.00	0.00–0.00
ω^2^_Ka2_	0.00	0.00	30.06	0.00	0.00–0.00
ω^2^_Ka3_	1.74	0.78	44.67	1.82	0.30–3.34
ω^2^_Tlag_	0.23	0.07	32.12	0.23	0.08–0.38

^a^ tv means for typical value. ^b^ means the quantitative correlation value between T_lag_ and gender (as a valid categorical covariate). ^c^ means the quantitative correlation value between K_a3_ and body surface area (BSA, as a valid covariate). V_c_/F, central compartment distribution volume; CL_c_/F, central compartment clearance; V_p_/F, peripheral compartment distribution volume; CL_p_/F, peripheral compartment clearance; K_a1_, first sequential absorption (dosing depot–depot 1) rate constant; K_a2_, second sequential absorption (depot 1–depot 2) rate constant; K_a3_, third sequential absorption (depot 2–central compartment) rate constant; T_lag_, lag time.

**Table 6:** Equation and parameter values for the rabeprazole pharmacodynamic model applying sigmoid E_max_ with baseline.

Model Equation	Parameters	Value	Standard Error	Relative Standard Error (%)	95% Confidence Interval
$E=E_{0}+(\frac{E_{\max}\times C^{\gamma }}{{{\mathrm{EC}}_{50}}^{\gamma }+C^{\gamma }}$)	E_0_	2.50	0.29	11.60	1.94–3.07
	E_max_	4.72	0.88	18.64	2.98–6.46
	γ	5.04	2.14	42.46	0.81–9.27
	EC_50_	51.58	5.20	10.08	41.29–61.87

E refers to the effect of increasing gastric pH. E_0_ refers to the basal pH effect in the stomach that occurs without rabeprazole. E_max_ refers to the maximal effect of increasing gastric pH by plasma rabeprazole level. EC_50_ is the concentration of plasma rabeprazole concentration required to achieve 50% of the E_max_. γ refers to the sigmoidicity factor related to the steepness of the profile. C refers to the concentration of rabeprazole in the plasma.

**Table 7:** Comparison of gastric pH changes between genders following oral administration of 10 mg rabeprazole enteric-coated tablets estimated using the rabeprazole population pharmacokinetic–pharmacodynamic model.

Population	AUEC ^a^ (h·pH Value)	Max Effect (pH)	Effect Time ^b^ (above pH 4, h)	Effect Duration Time ^c^ (above pH 4, h)
Total				
5% of 50th percentiles	25.90	4.65	3.75–4.20	0.45
50% of 50th percentiles	31.22	6.41	2.60–5.07	2.47
95% of 50th percentiles	36.72	7.02	2.04–5.60	3.56
Male				
5% of 50th percentiles	27.11	5.52	2.55–4.04	1.49
50% of 50th percentiles	32.73	6.87	2.02–4.81	2.79
95% of 50th percentiles	39.45	7.14	1.13–5.49	4.36
Female				
5% of 50th percentiles	23.25	3.08	- ^d^	0.00
50% of 50th percentiles	29.90	5.95	3.20–5.29	2.09
95% of 50th percentiles	39.61	7.07	2.40–6.42	4.02

^a^ area under the effect curve (pH change according to rabeprazole exposure time); ^b^ refers to the estimated time that pH in the stomach remains above 4. ^c^ refers to the entire duration that the pH in the stomach is maintained above 4. ^d^ not applicable.

## Data Availability

All data and related materials are accessible in this manuscript and Supplementary Materials.

## Supplementary Materials

The following supporting information can be downloaded at: https://www.mdpi.com/article/10.3390/biomedicines11113021/s1, Supplementary Information S1: Determination of biochemical parameters; Supplementary Information S2: Subjects; Supplementary Information S3: Clinical trial design and sampling; Supplementary Information S4: Determination of rabeprazole plasma concentrations; Supplementary Information S5: Non-compartment analysis; Supplementary Information S6: Model qualification tools; Supplementary Information S7: Population pharmacokinetic model equations for rabeprazole enteric-coated tablets; Table S1: Demographic information of the health of Korean subjects orally administered a single 10 mg rabeprazole enteric-coated tablet (*n* = 45); Table S2: Gender comparison of demographic information for healthy Korean subjects (*n* = 45) who received a single oral administration of rabeprazole 10 mg enteric-coated tablet; Figure S1: Boxplot comparison of male and female pharmacokinetic parameter values according to oral exposure to a 10 mg rabeprazole enteric-coated tablet; Figure S2: Boxplot comparison of pharmacokinetic parameter values between males and females with confirmed statistical significance according to oral exposure to a 10 mg rabeprazole enteric-coated tablet; Figure S3: Boxplot comparison between genders of pharmacokinetic parameter values estimated based on body weight-normalized plasma concentration values following oral administration of a 10 mg rabeprazole enteric-coated tablet; Figure S4: Boxplot comparison between genders of maximum plasma concentration values determined based on plasma concentration values before and after body weight normalization following oral administration of a 10 mg rabeprazole enteric-coated tablet; Figure S5: Goodness-of-fit plots of the final pharmacokinetic model for rabeprazole.
