# Supplementary material for: Exploring Differences in Pharmacometrics of Rabeprazole between Genders via Population Pharmacokinetic–Pharmacodynamic Modeling
Source: Biomedicines. 2023 Nov 10;11(11):3021. doi: 10.3390/biomedicines11113021 (PMC10669052; doi:10.3390/biomedicines11113021)
Supplement: Supplementary file 1 [file biomedicines-11-03021-s001.zip › biomedicines-2698856-supplementary.pdf]

Exploring differences in pharmacometrics of rabeprazole between genders through population pharmacokinetic–pharmacodynamic modeling

Supplementary Information, Tables, and Figures (with captions)

Seung-Hyun Jeong [1,2,a], Ji-Hun Jang [1,a], Yong-Bok Lee [3,*]

[1] *College of Pharmacy, Sunchon National University, 255 Jungang-ro, Suncheon-Si, Jeollanam-do, 57922, Republic of Korea*

[2] *College of Pharmacy and Research Institute of Life and Pharmaceutical Sciences, Sunchon National University, Suncheon-Si 57922, Republic of Korea*

[3] *College of Pharmacy, Chonnam National University, 77 Yongbong-ro, Buk-gu, Gwangju, 61186, Republic of Korea*

[a] Both authors contributed equally to this work.

* Corresponding author:
Prof. Yong Bok Lee
College of Pharmacy, Chonnam National University, 77 Yongbong-ro, Buk-gu, Gwangju, 61186, Republic of Korea
Tel.: 82-62-530-2931; Fax: 82-62-530-0106
E-mail: leeyb@chonnam.ac.kr

Supplementary Information 1

Determination of biochemical parameters

Blank plasma samples obtained immediately before rabeprazole administration (as 0 h) were used to analyze the biochemical parameters. The types of biochemical parameters included total proteins, albumin, globulin, alkaline phosphatase (ALP), aspartate transaminase (AST), alanine transaminase (ALT), cholesterol, total bilirubin, glucose, blood urea nitrogen, gamma-glutamyl transferase, creatinine, creatinine clearance (CrCL), and glomerular filtration rate (GFR). Hematological tests included white blood cells, red blood cells, platelets, neutrophils, lymphocytes, and eosinophil counts, and hemoglobin and hematocrit. Determination of clinical biochemical parameter values was performed by serological analysis through a dry automated analyzer. The analytical instrument used was a VITROS Microslide (Ortho Clinical Diagnostics, NJ, USA), which was operated by reflectance spectrophotometry. CrCL was calculated based on the Cockcroft–Gault equation, which is: $((140 - \text{age}) \times \text{body weight (kg)}/\text{serum creatinine (mg/dL)} \times 72)$. GFR was calculated using the modification-of-diet-in-renal-disease formula, which is: $186.3 \times (\text{serum creatinine (mg/dL)}^{-1.154} \times \text{age (year)}^{-0.203})$. Body mass index (BMI) and body surface area (BSA) were calculated based on measurement information relating to the body weight and height of the individuals. BMI was calculated based on the Kaup index, which is: (body weight (kg)/height^2 (m^2)]. BSA was calculated based on the Mosteller formula, which is: $\sqrt{(\text{height (cm)} \times \text{body weight (kg)}/3600)}$.

Supplementary Information 2

Subjects

A total of 75 subjects were recruited to conduct this clinical trial, of which, 45 met the inclusion criteria and participated in the final trial. Inclusion criteria were as follows: Age 19 years or older at screening; obesity index BMI within the range of 18–30 kg/m^2; body weight greater than 50 kg and 45 kg for men and women, respectively; no clinically significant congenital or chronic diseases and no pathological symptoms or findings as a result of medical examination; diagnostic tests (blood and urine tests) and electrocardiogram results were within normal values. The exclusion criteria were as follows: Taking drugs that induce or inhibit drug-metabolizing enzymes, such as barbiturates, within 30 days of the test start date, or taking drugs that may affect the test within 10 days; a history of gastrointestinal resection, which may affect drug absorption; excessive drinking within 1 month prior to the test start date; hypersensitivity to the test drug or its components (especially benzimidazole type). All 45 subjects who participated in the test underwent a thorough health examination (vital signs, physical examination, electrocardiogram, hematology test, blood chemistry test, urine test, serology test, etc.). No clinically significant abnormal values or findings were identified, and no concomitant medications were administered. All subjects provided written informed consent before participating in the clinical trial. This clinical study was conducted in accordance with the Declaration of Helsinki as implemented in the Good Clinical Practice guidelines. The clinical trial was conducted at the Bumin Clinical Trial Center (Seoul, Republic of Korea) from April 2, 2022, to April 25, 2022.

Supplementary Information 3

Clinical trial design and sampling

This bioequivalence investigation was performed as randomized, single-dose, open-label, crossover, and two-way studies (with a washout period of 7 days). Prior to the clinical trial, subjects had a heparin-locked (150 unit/mL) JELCO 22G angio-catheter (Smiths Medical, Minneapolis, MN, USA) installed into a vein on the arm or back of the hand, and 6 mL of blank blood was collected. Then, all subjects were orally administered a single 10 mg rabeprazole enteric-coated tablet along with 150 mL of water. Subjects had fasted for more than 10 h before administration. Blood sampling from subjects was performed at a total of 13 specific time points post-dose: Before administration (0 h) and at 1, 2, 2.5, 3, 3.5, 4, 4.5, 5, 5.5, 6, 7, 8, and 10 h after oral administration. The sampling method was as follows: To completely remove the heparinized saline solution remaining in the venous catheter during blood collection, approximately 1 mL of blood was collected and discarded each time, and approximately 6 mL of blood was collected and placed in sodium heparinized vacutainer tubes (BD, Franklin Lakes, NJ, USA) with the subject management number and blood collection time written on them. After each blood draw, a saline solution for injection (containing heparin for injection) was administered to prevent the blood remaining in the intravenous catheter from clotting. The collected blood was centrifuged at 3,000 $\times g$ for 10 min in a centrifuge set at 4 °C, and then approximately 1 mL of plasma was taken, transferred to an Eppendorf tube (Eppendorf, Hamburg, Germany), and stored at −80 °C until analysis.

Supplementary Information 4

Determination of rabeprazole plasma concentrations

The rabeprazole concentrations in the plasma were determined by ultra-high-performance liquid chromatography coupled using a mass spectrometer, which was established and validated in previous studies. Briefly, plasma samples were pretreated through protein precipitation using acetonitrile, and then, selectively separated on an Imtakt Unison UK-C_{18} (3 μm, 2.0 × 100 mm) column using 5 mM aqueous ammonium formate and acetonitrile as mobile phases. The mobile phase composition ratio of 5 mM aqueous ammonium formate and acetonitrile was isocratically eluted at 55:45 (v/v). Quantification of rabeprazole was performed by positive electrospray using multiple reaction monitoring modes. Rabeprazole-d_4 was used as an internal standard (IS), and the mass transitions of rabeprazole and IS were 360.10 → 242.10 and 364.20 → 242.10, respectively. The retention times for rabeprazole and IS were 1.39 and 1.38 min, respectively. The lower quantification limit was 1 ng/mL, and the linear calibration curve was secured using a coefficient of determination of 0.99 or higher, up to the concentration range of 100 ng/mL. If the rabeprazole concentration in the sample exceeded the upper quantification limit of 100 ng/mL, the sample was diluted with blank plasma and re-analyzed within the calibration curve. The intra- and inter-day precision and accuracy of the method were all within 15%, and no significant carryover or matrix effect was identified.

Supplementary Information 5

Non-compartment analysis

Basic pharmacokinetic parameters calculations were performed for rabeprazole by non-compartment analysis (NCA) using Phoenix WinNonlin software (version 8.3, Pharsight, Certara Inc.). The area under the curve from 0 h to infinite (AUC_{inf}) was calculated as the sum of AUC_{all} and C_{last}/k, where C_{last} is the final measured rabeprazole plasma concentration and k is the elimination rate constant at the terminal phase. AUC_{all} was calculated using a linear trapezoidal rule from 0 to t h after oral administration of the rabeprazole 10 mg enteric-coated tablet, where t is the time in C_{last}. The area under the first-order moment curve from 0 h to infinite ($AUMC_{inf}$) was calculated as the sum of $AUMC_{all}$ and $C_{last} \cdot T_{last}/k$ and T_{last}/k^2, where T_{last} is the time in C_{last}. $AUMC_{all}$ was calculated using a linear trapezoidal rule from 0 to t h (as the area of the graph under the product of time and rabeprazole plasma concentration over time) after oral administration of the rabeprazole 10 mg enteric-coated tablet. The mean residence time (MRT) was obtained as the ratio of $AUMC_{inf}$ and AUC_{inf}. The half-life ($T_{1/2}$) was calculated as 0.693/k, and the volume of the distribution (V/F) was calculated as dose/k · AUC_{inf}. The clearance (CL/F) was calculated by dividing the rabeprazole dose by AUC_{inf}, where F is the bioavailability of oral administration. The peak plasma drug concentration (C_{max}) and time to reach C_{max} (T_{max}) were determined from the plasma rabeprazole concentration–time curves for each individual after oral administration of the rabeprazole 10 mg enteric-coated tablet.

Supplementary Information 6

Model qualification tools

The goodness-of-fit (GOF) was confirmed using diagnostic scatter plots as follows: (A) Population-predicted concentrations (PRED) versus observed concentrations (DV), (B) individual-predicted concentrations (IPRED) versus DV, (C) PRED versus conditional weighted residuals (CWRES), (D) time after dose (IVAR) versus CWRES, (E) quantile–quantile (QQ) plot of the CWRES components, and (F) QQ plot of the weighted residuals (WRES) components. A visual predictive check (VPC) of the final established model was performed using the VPC option in Phoenix NLME (version 8.4). The number of simulations for the VPC was 1000. The IVAR–DV concentration data were graphically superimposed on the median values and the 5th and 95th percentiles of the IVAR-simulated concentration profiles. If the DV concentration data were approximately distributed within the 95th and 5th prediction intervals, the model was expected to be precise. By using non-parametric bootstrap analysis, the stability of the final model was confirmed. For this, the bootstrap option in Phoenix NLME was used. A total of 1000 replicates were generated by the repeated random sampling with replacement from the original dataset. The estimated parameter values, such as the standard errors (SE; including confidence interval (CI)) and medians from the bootstrap procedure, were compared to those estimated from the original dataset.

Supplementary Information 7

Population pharmacokinetic model equations for rabeprazole enteric-coated tablets

The formula for the final established population pharmacokinetic model (based on the results of the pharmacokinetic profile obtained after a single oral dose of a rabeprazole 10 mg enteric-coated tablet) parameters of rabeprazole were as follows:

$$\frac{Vc}{F} = tv\ \frac{Vc}{F} \cdot \exp(\eta_{Vc/F})$$

$$\frac{CLc}{F} = tv\ \frac{CLc}{F} \cdot \exp(\eta_{CLc/F})$$

$$\frac{Vp}{F} = tv\ \frac{Vp}{F}$$

$$\frac{CLp}{F} = tv\ \frac{CLp}{F}$$

$$K_{a1} = tv\ K_{a1} \cdot \exp(\eta_{Ka1})$$

$$K_{a2} = tv\ K_{a2} \cdot \exp(\eta_{Ka2})$$

$$K_{a3} = tv\ K_{a3} \cdot (BSA/median\ BSA)\text{^}dK_{a3}dBSA \cdot \exp(\eta_{Ka3})$$

$$T_{lag} = tv\ T_{lag} \cdot (1 + dT_{lag}dGender \cdot (\text{if female} = 1 \text{ and male} = 0)) \cdot \exp(\eta_{Tlag})$$

where tv meant typical parameter values, median BSA meant the median of body surface area (BSA) levels in the observed population, and $dK_{a3}dBSA$ meant the degree of correlation between K_{a3} and BSA. The $dT_{lag}dGender$ meant the degree of correlation between T_{lag} and gender. V_c/F, central compartment distribution volume; CL_c/F, central compartment clearance; V_p/F, peripheral compartment distribution volume; CL_p/F, peripheral compartment clearance;

K_{a1}, first sequential absorption (dosing depot-to-depot 1) rate constant; K_{a2}, second sequential absorption (depot 1-to-depot 2) rate constant; K_{a3}, third sequential absorption (depot 2-to-central compartment) rate constant; T_{lag}, lag-time. The inter-individual variabilities in pharmacokinetic parameters of rabeprazole were evaluated using an exponential error model, as shown in the following equation: $P_i = P_{tv} \cdot \exp(\eta_i)$, where η_i is the random variable for the ith individual, which was normally distributed with a mean of 0 and variance ω^2, P_i is the parameter value of the ith individual, and P_{tv} is the typical population parameter value.

Table S1. Demographic information of the health of Korean subjects orally administered a single 10 mg rabeprazole enteric-coated tablet (*n* = 45).

**Demographic**	**Unit**	**Value (mean ± standard deviation)**	**Value range (median)**
Age	year	32.31 ± 8.40	20.00–49.00 (31.00)
Height	cm	167.83 ± 8.59	150.50–183.90 (168.10)
Body weight	kg	66.66 ± 11.98	45.60–94.10 (66.00)
Body mass index	kg/m^2	23.54 ± 2.93	18.20–29.50 (23.30)
Body surface area	m^2	1.76 ± 0.19	1.41–2.16 (1.75)
White blood cell count	$\times 10^3$ count/μL	6.75 ± 1.78	3.86–11.20 (6.54)
Red blood cell count	$\times 10^6$ count/μL	4.65 ± 0.43	3.85–5.46 (4.72)

Hemoglobin	g/dL	14.41 ± 1.60	11.80–17.40 (14.80)
Hematocrit	%	43.66 ± 4.26	37.40–50.10 (44.80)
Platelet count	× 10^3 count/μL	253.44 ± 49.26	112.00–455.00 (250.00)
Neutrophils	%	58.54 ± 9.84	39.00–76.60 (59.00)
Lymphocytes	%	31.26 ± 8.18	15.80–49.10 (30.50)
Eosinophils	%	2.78 ± 2.29	0.50–10.60 (2.10)
Absolute neutrophil count	count/mm^3	4030.71 ± 1556.10	1903.00–8389.00 (3523.00)
Blood urea nitrogen	mg/dL	12.68 ± 3.20	6.80–21.20 (12.80)

Creatinine	mg/dL	0.76 ± 0.17	0.50–1.20 (0.80)
Creatinine clearance	mL/min	123.70 ± 24.25	95.21–188.89 (115.20)
Estimated glomerular filtration rate	mL/min/1.73 m²	106.89 ± 18.25	67.10–157.30 (102.80)
Total protein	g/dL	7.40 ± 0.30	6.90–8.10 (7.40)
Albumin	g/dL	4.64 ± 0.22	4.30–5.20 (4.60)
Alkaline phosphatase	U/L	64.31 ± 16.31	35.00–108.00 (64.00)
Aspartate transaminase	U/L	24.00 ± 6.68	14.00–47.00 (22.00)
Alanine transaminase	U/L	24.02 ± 12.17	12.00–66.00 (21.00)

Gamma-glutamyl transpeptidase	U/L	19.27 ± 11.80	6.00–62.00 (17.00)
Total bilirubin	mg/dL	0.98 ± 0.38	0.30–2.10 (0.90)
Glucose	mg/dL	84.93 ± 6.38	70.00–100.00 (86.00)
Total cholesterol	mg/dL	184.09 ± 29.91	127.00–262.00 (181.00)

Table S2. Gender comparison of demographic information for healthy Korean subjects (n = 45) who received a single oral administration of rabeprazole 10 mg enteric-coated tablet.

**Demographic**	**Unit**	**Value (mean ± standard deviation)**	**Value (mean ± standard deviation)**	***P* value**
		**Male (*n* = 24)**	**Female (*n* = 21)**	
Age	year	33.29 ± 7.44	31.19 ± 9.44	4.09×10^{-1}
Height	cm	173.57 ± 6.36	161.27 ± 5.58	2.14×10^{-8} *
Body weight	kg	74.05 ± 9.90	58.21 ± 7.90	5.62×10^{-7} *
Body mass index	kg/m²	24.55 ± 2.67	22.39 ± 2.84	1.18×10^{-2} *
Body surface area	m²	1.89 ± 0.15	1.61 ± 0.12	3.05×10^{-8} *

White blood cell count	$\times 10^3$ count/μL	6.94 ± 1.84	6.53 ± 1.73	4.49×10^{-1}
Red blood cell count	$\times 10^6$ count/μL	4.95 ± 0.29	4.31 ± 0.28	2.27×10^{-9} *
Hemoglobin	g/dL	15.66 ± 0.83	12.99 ± 0.90	2.80×10^{-13} *
Hematocrit	%	47.08 ± 2.14	39.76 ± 2.17	1.56×10^{-14} *
Platelet count	$\times 10^3$ count/μL	238.08 ± 38.05	271.00 ± 55.39	2.81×10^{-2} *
Neutrophils	%	57.04 ± 10.31	60.26 ± 9.23	2.78×10^{-1}
Lymphocytes	%	31.90 ± 7.98	30.51 ± 8.53	5.75×10^{-1}
Eosinophils	%	3.49 ± 2.78	1.97 ± 1.16	1.96×10^{-2} *
Absolute neutrophil	count/mm^3	4069.17 ± 1701.64	3986.76 ± 1411.90	8.62×10^{-1}

count				
Blood urea nitrogen	mg/dL	14.03 ± 2.94	11.14 ± 2.81	1.71×10^{-3} *
Creatinine	mg/dL	0.90 ± 0.11	0.61 ± 0.07	7.11×10^{-13} *
Creatinine clearance	mL/min	124.18 ± 24.88	123.14 ± 24.12	8.88×10^{-1}
Estimated glomerular filtration rate	mL/min/1.73 m²	98.51 ± 14.09	116.47 ± 18.00	5.24×10^{-4} *
Total protein	g/dL	7.42 ± 0.26	7.37 ± 0.34	6.18×10^{-1}
Albumin	g/dL	4.71 ± 0.20	4.56 ± 0.22	1.83×10^{-2} *
Alkaline phosphatase	U/L	72.92 ± 15.30	54.48 ± 11.22	4.31×10^{-5} *

Aspartate transaminase	U/L	25.63 ± 8.36	22.14 ± 3.32	6.97×10^{-2}
Alanine transaminase	U/L	27.92 ± 14.28	19.57 ± 7.26	1.66×10^{-2} *
Gamma-glutamyl transpeptidase	U/L	24.13 ± 13.47	13.71 ± 6.07	1.73×10^{-3} *
Total bilirubin	mg/dL	1.04 ± 0.45	0.91 ± 0.28	2.51×10^{-1}
Glucose	mg/dL	85.46 ± 6.36	84.33 ± 6.51	5.61×10^{-1}
Total cholesterol	mg/dL	184.13 ± 31.38	184.05 ± 28.91	9.93×10^{-1}

*$p < 0.05$ between male pharmacokinetic parameters.

Supplementary figure captions

Figure S1. Boxplot comparison of male and female pharmacokinetic parameter values (A, AUC_{all}; B, AUC_{inf}; C, CL/F; D, $T_{1/2}$; E, MRT; F, V_d/F), according to oral exposure to a 10 mg rabeprazole enteric-coated tablet. AUC_{all}: area under the curve from 0 to observed (t) time after administration; AUC_{inf}: area under the curve from 0 to infinity time after administration. CL: clearance; $T_{1/2}$: half-life; MRT: mean residence time; V_d: volume of distribution; F: oral bioavailability.

Figure S2. Boxplot comparison of pharmacokinetic parameter values (A, T_{max}; B, T_{lag}) between males and females with confirmed statistical significance ($*p < 0.05$ between male pharmacokinetic parameters), according to oral exposure to a 10 mg rabeprazole enteric-coated tablet. T_{max}: time to reach the maximum plasma concentration; T_{lag}: lag time in absorption.

Figure S3. Boxplot comparison between genders of pharmacokinetic parameter values (A, AUC_{all}; B, AUC_{inf}; C, wCL/F; D, wV_d/F) estimated based on body weight-normalized plasma concentration values following oral administration of a 10 mg rabeprazole enteric-coated tablet. AUC_{all}: area under the curve from 0 to observed (t) time after administration; AUC_{inf}: area under the curve from 0 to infinity time after administration; wCL: clearance estimated based on plasma rabeprazole concentrations normalized to body weight; wV_d: volume of distribution estimated based on plasma rabeprazole concentrations normalized to body weight; F: oral bioavailability.

Figure S4. Boxplot comparison between genders of maximum plasma concentration (C_{max}) values determined based on plasma concentration values before (A) and after (B) body weight normalization following oral administration of a 10 mg rabeprazole enteric-coated tablet. $*p < 0.05$ between male C_{max} values.

Figure S5. Goodness-of-fit plots of the final pharmacokinetic model for rabeprazole. (A) Population-predicted concentrations (PRED, natural log scale, ng/mL) against observed plasma concentrations (DV, natural log scale, ng/mL), (B) Individual-predicted concentrations (IPRED, natural log scale, ng/mL) against DV, (C) PRED against conditional weighted residuals (CWRES), (D) Time (IVAR, h) against CWRES, and (E) quantile–quantile (QQ) plot of CWRES components. Empty points in the graphs (A–E) represent each data value predicted or estimated by the model.

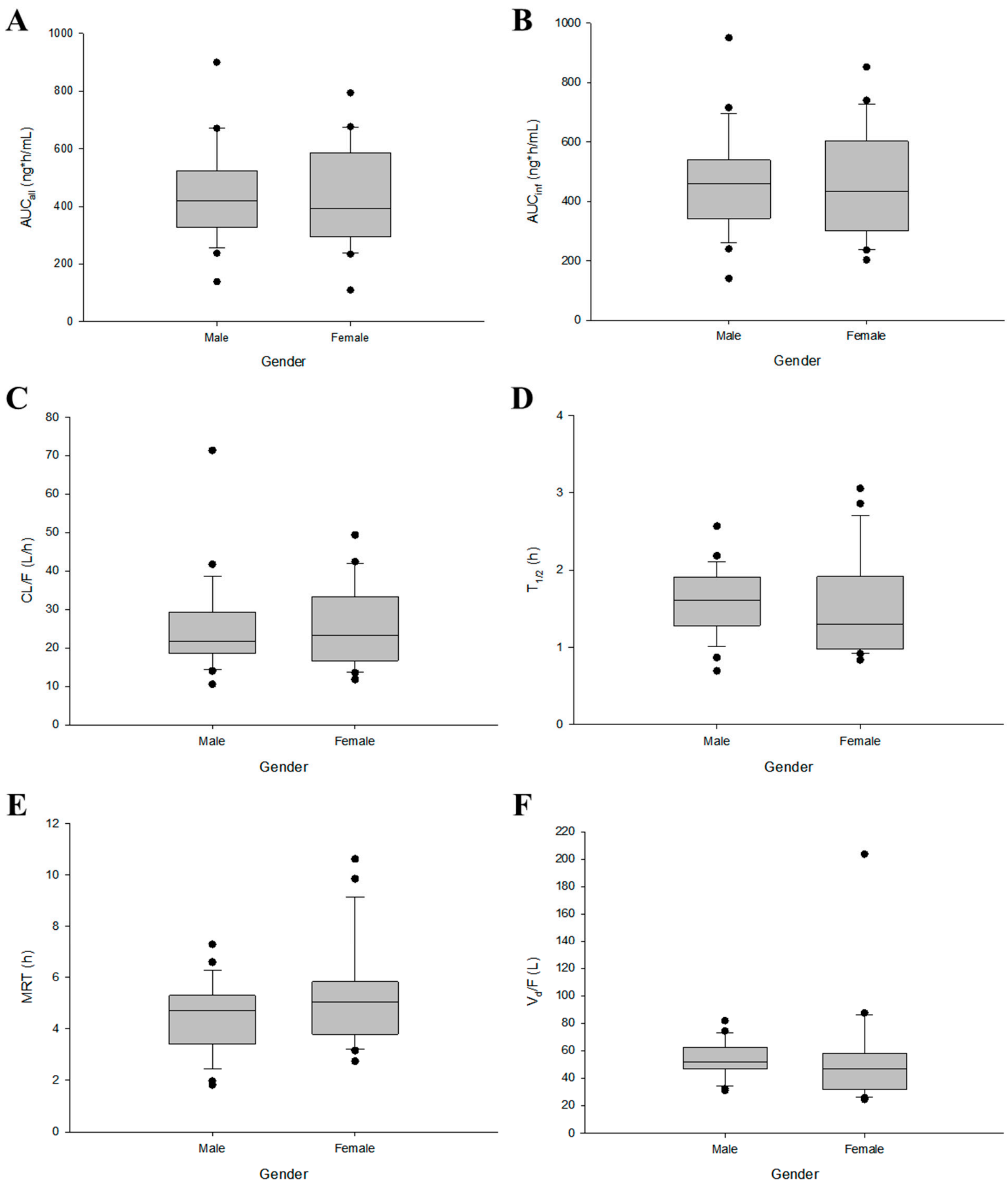
A
AUC_all (ng*h/mL)
Male
Female
Gender
B
AUC_inf (ng*h/mL)
Male
Female
Gender
C
CL/F (L/h)
Male
Female
Gender
D
T_1/2 (h)
Male
Female
Gender
E
MRT (h)
Male
Female
Gender
F
V_d/F (L)
Male
Female
Gender

Figure S1

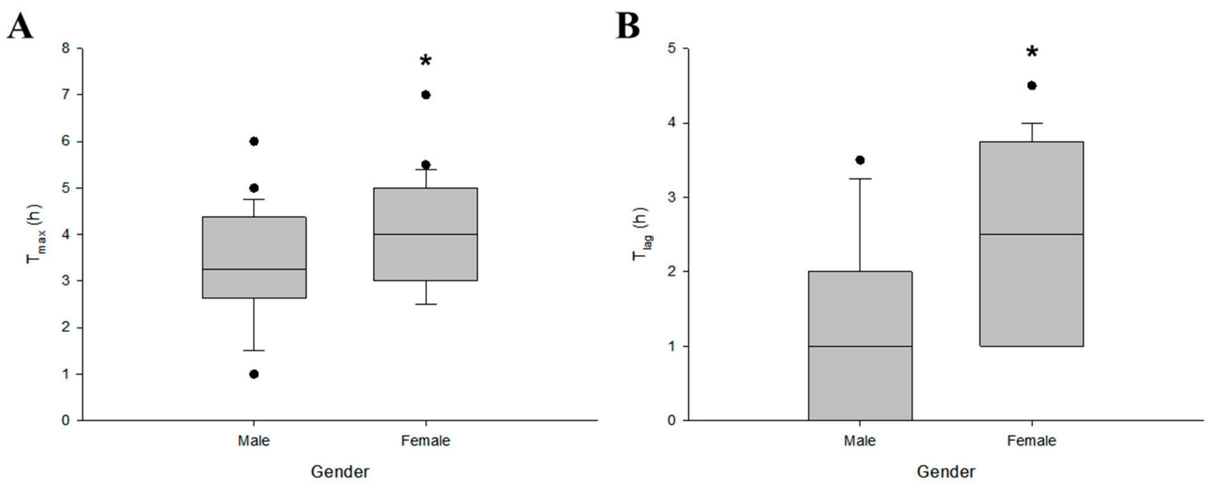
A
B
8
7
6
5
4
3
2
1
0
T_{max} (h)
*
Male
Female
Gender
5
4
3
2
1
0
T_{lag} (h)
*
Male
Female
Gender

Figure S2

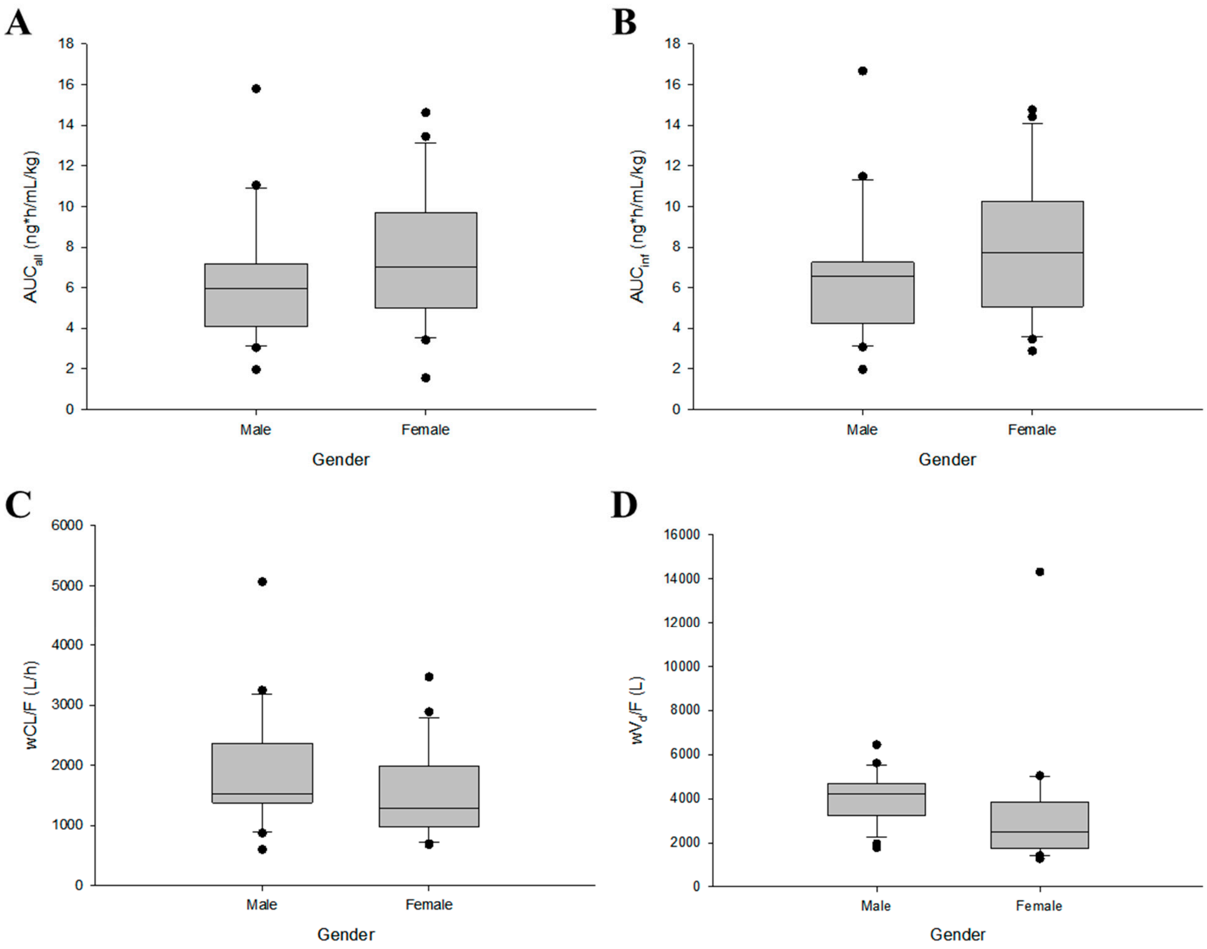

187

188 **Figure S3**

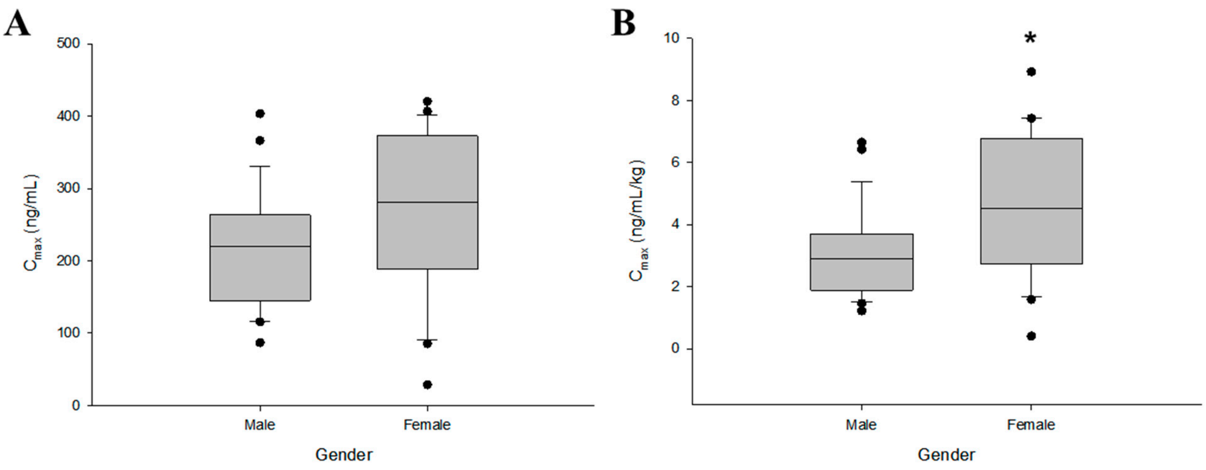
A
500
400
300
200
100
0
Cmax (ng/mL)
Male
Female
Gender
B
10
8
6
4
2
0
*
Cmax (ng/mL/kg)
Male
Female
Gender

Figure S4

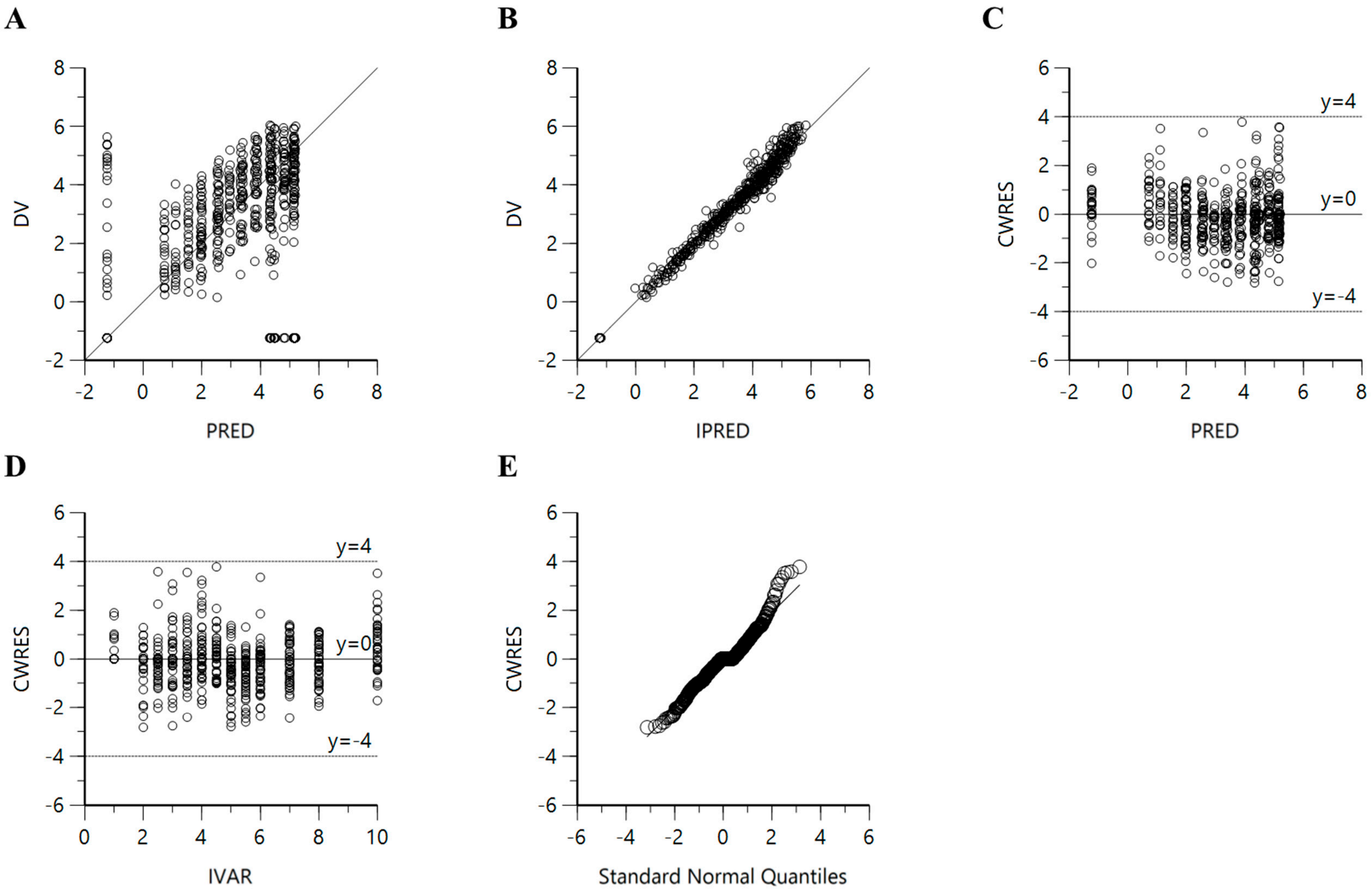
A
B
C
D
E
DV
PRED
IPRED
CWRES
IVAR
Standard Normal Quantiles
y=4
y=0
y=-4

Figure S5
